# Supplementary figures and images for: Activation of the ciliary kinase CDKL5 is mediated by the cyclin-dependent kinase CDK20/LF2 to control flagellar length
Source: PLoS Biol. 2025 Dec 12;23(12):e3003560. doi: 10.1371/journal.pbio.3003560 (PMC12711092; doi:10.1371/journal.pbio.3003560)

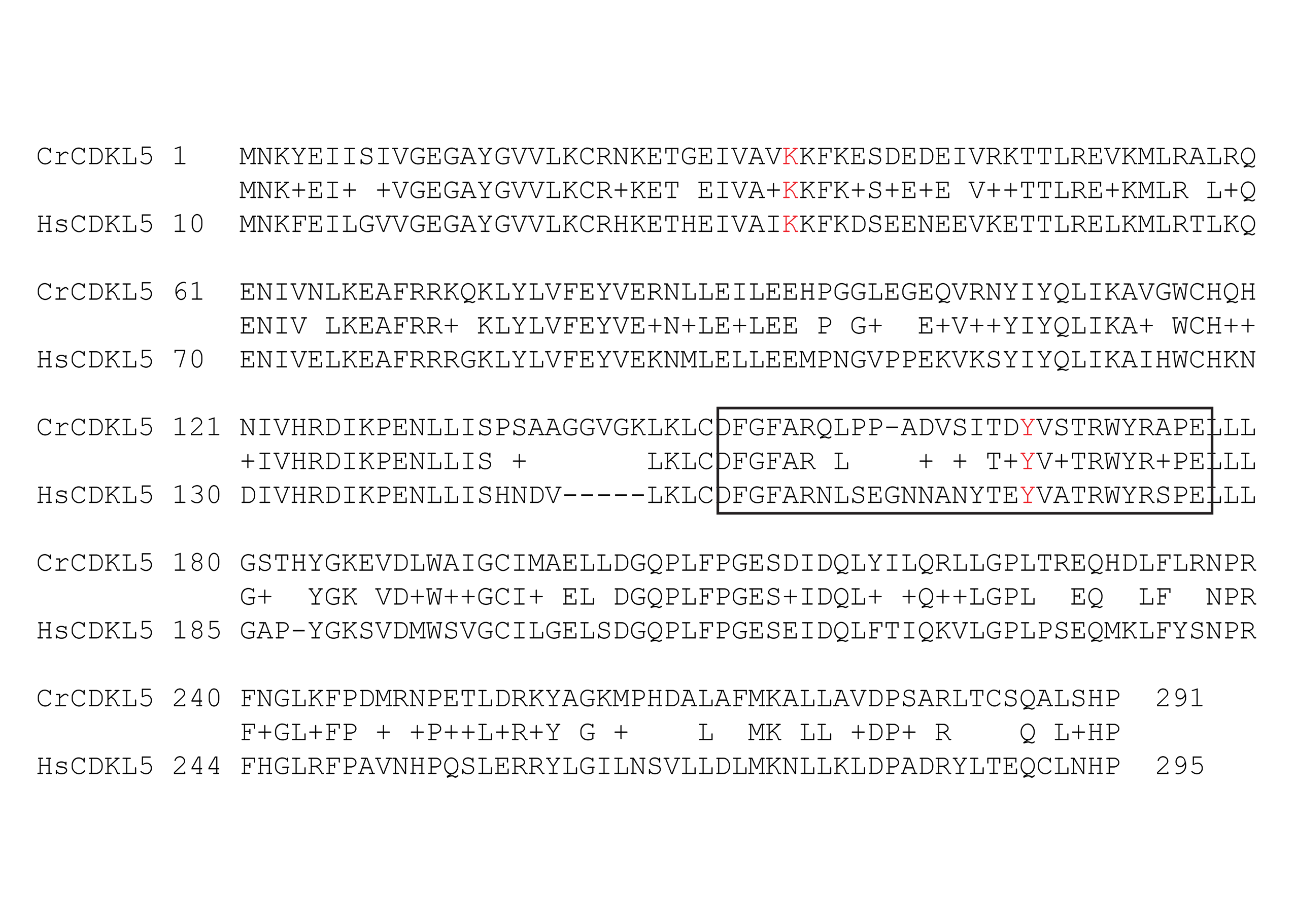

Supplement: S1 Fig — Box marks the activation loop. Red K is the active site lysine and red Y is the activation loop tyrosine. (TIF) [file pbio.3003560.s001.tif]

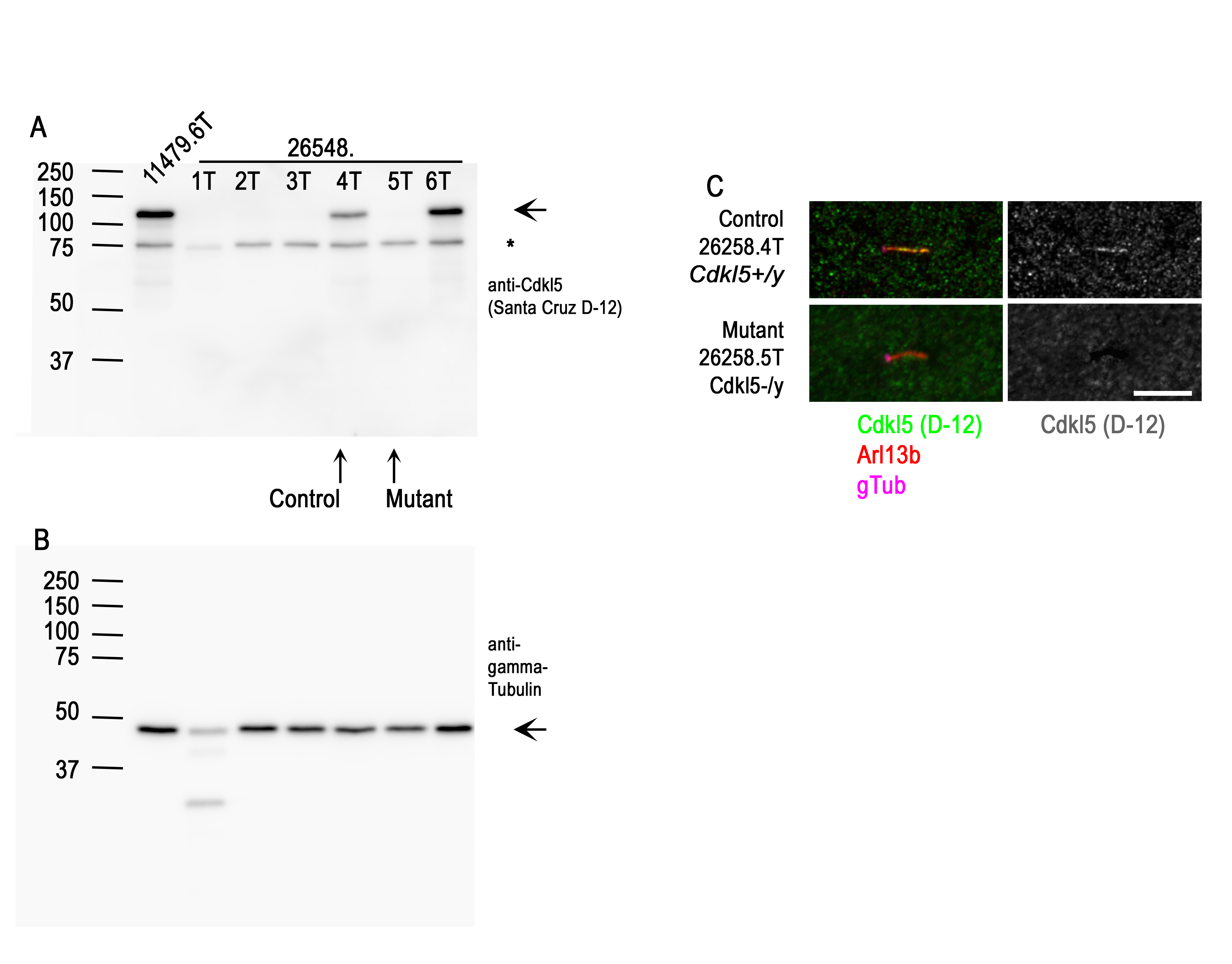

Supplement: S2 Fig — (A, B) Western blots of MEF extracts probed with CDKL5 Santa Cruz monoclonal antibody clone D-12 (A) and γ-tubulin antibodies (B). Arrow marks the expected band. *marks a nonspecific band. 11479.6T is the control line used for generation of CRISPR knockouts. 26548.1-.6T are lines derived from embryos of the B6.129(FVB)-Cdkl5tm1.1Joez/J mouse. 26548.4T (+/y) (Control) and 26548.5T (−/y) (Mutant) were used for further work. (C) Immunofluorescence of wild-type and Cdkl5-mutant fibroblasts labeled with CDKL5 Santa Cruz monoclonal antibody clone D-12 (green in left panel, gray in right panel), Arl13b (red in left panel), and γ-tubulin (pink in left panel). Scale bar is 5 microns. (TIF) [file pbio.3003560.s002.tif]

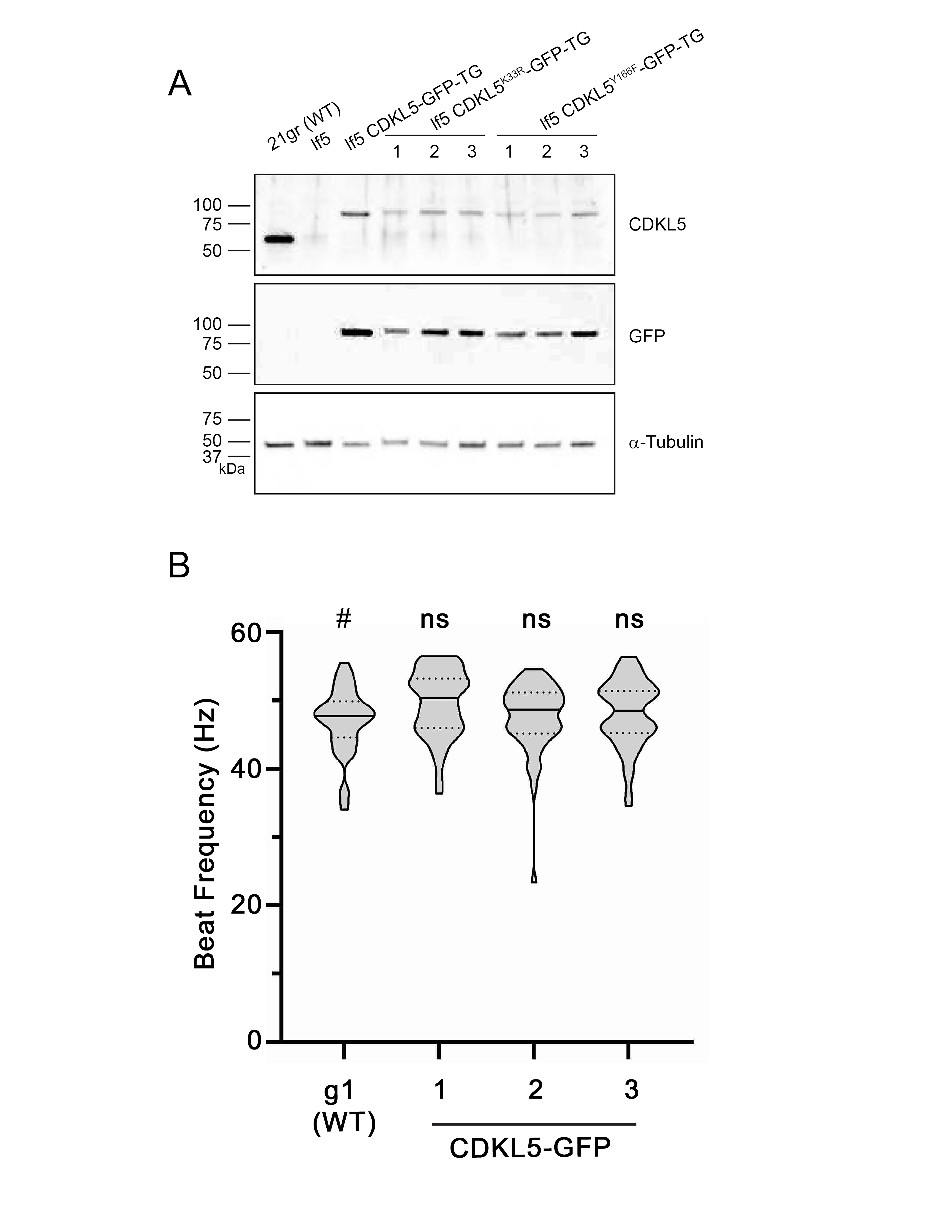

Supplement: S3 Fig — (A) Western blots of flagella samples from wild-type (21gr), lf5, lf5 CDKL5-GFP-TG, three different transformants of lf5 CDKL5K33R-GFP-TG, and three different transformants of lf5 CDKL5Y166F-GFP-TG cells were probed with anti-CDKL5 or anti-GFP to reveal CDKL5 proteins. The same set of samples was diluted 1:25 and probed with anti-α-tubulin as a loading control. (B) Beat frequency of wild-type cells (g1) and three independent lines (CDKL5-GFP) created by CRISPR in the g1 background. Fifty cells were measured for each cell line. ns: not significant as compared to wild-type (#) by one-way ANOVA with Tukey’s multiple comparisons post-hoc test. Violin plots show median (solid line) and quartiles (dashed lines). Underlying data can be found in S1 Data. (TIF) [file pbio.3003560.s003.tif]

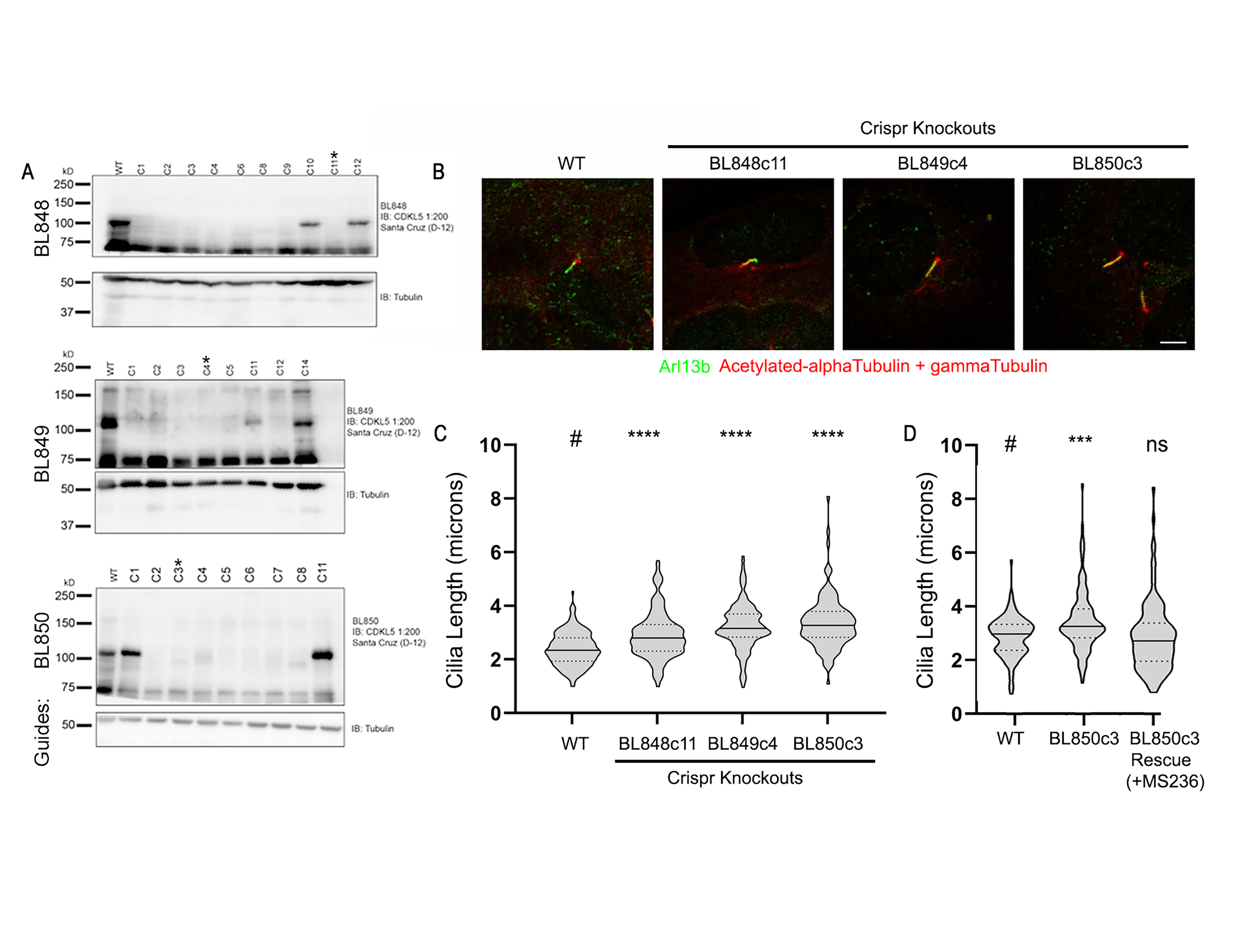

Supplement: S4 Fig — (A) Mouse embryonic fibroblast line 11479.6T was transfected with lentiviral guide constructs BL848, BL849, and BL850. After drug selection, the cells were single-cell sorted into 96 well dishes and then screened by western blotting with a Cdkl5 antibody (Santa Cruz D-12) using a γ-tubulin antibody as loading control. Asterisks (*) mark cell lines that were subsequently analyzed. (B) Control and the knockout cells were stained for cilia (acetylated α-tubulin and γ-tubulin in red, Arl13b in green). Scale bar, 5 µm. Z projection of slices taken at 0.37-µm intervals. (C) Quantification of cilia length in the cells described in B. n > 100 for each condition. ****p ≤ 0.0001 as compared to control (#) by one-way ANOVA with Tukey’s multiple comparisons post-hoc test. Violin plots show median (solid line) and quartiles (dashed lines). Underlying data can be found in S1 Data. (D) Rescue. Quantification of cilia length in the wild-type, BL850c3, and BL850c3 rescued with wild-type CDKL5 mutated to resist the guide RNA (MS236). n > 100 for each condition. ***p ≤ 0.001, ns: not significant as compared to control (#) by one-way ANOVA with Tukey’s multiple comparisons post-hoc test. Violin plots show median (solid line) and quartiles (dashed lines). Underlying data can be found in S1 Data. (TIF) [file pbio.3003560.s004.tif]

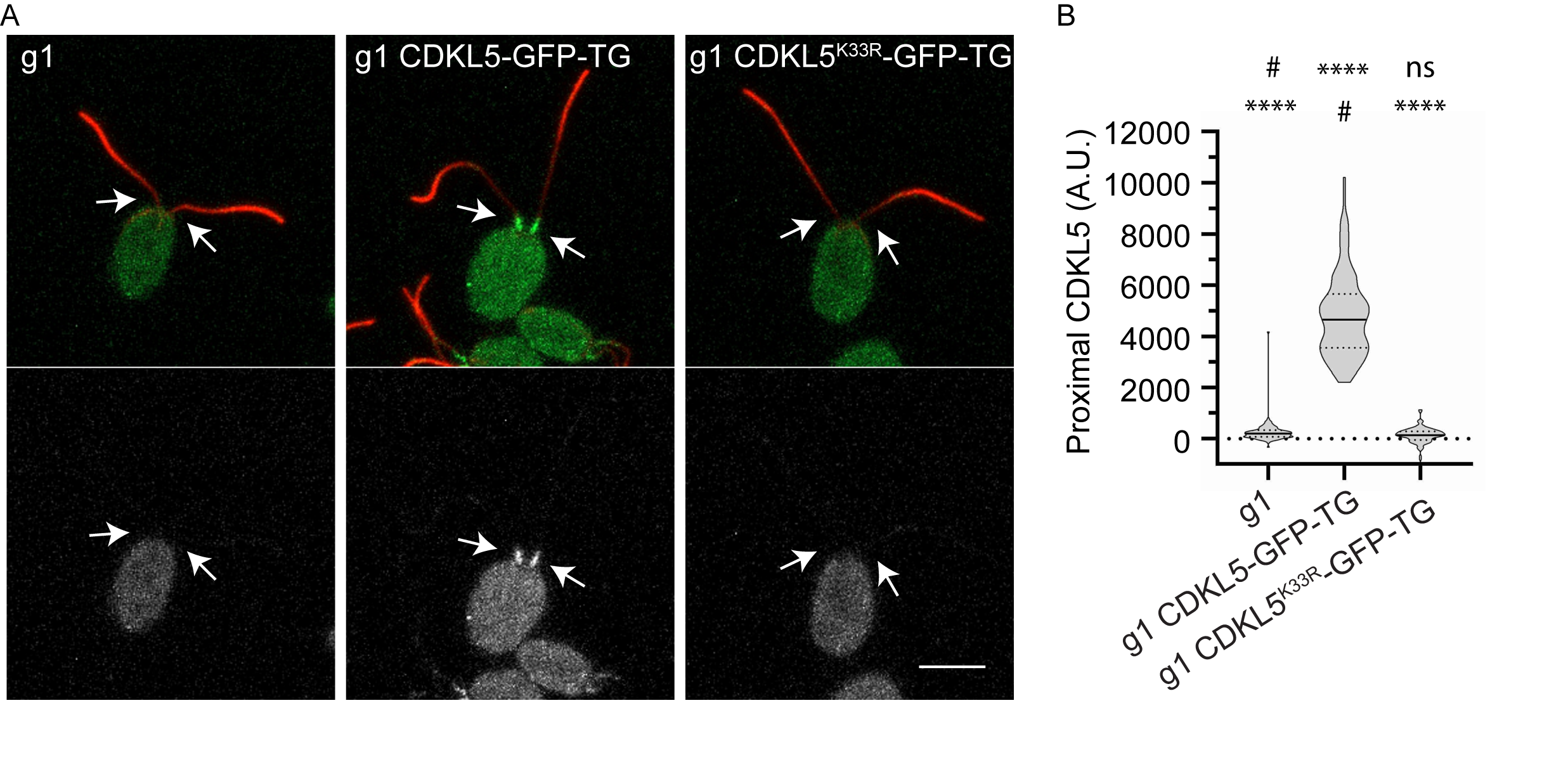

Supplement: S5 Fig — (A) Wild-type (g1) cells untransformed or expressing CDKL5-GFP or CDKL5K33R-GFP were stained for flagella (acetylated tubulin, red) and CDKL5 (GFP, green in top panels, gray in bottom panels). The cell bodies of all strains are green (or gray) due to autofluorescence. Wild-type CDKL5-GFP concentrates at the basal end of the flagellar shaft (arrows) while CDKL5K33R-GFP does not, similar to what was observed when these constructs were expressed in lf5 mutant cells (Fig 7). (B) Quantification of the CDKL5 pool at the base of the flagellar shaft. The quantification tools of ImageJ were used to measure fluorescence intensity in an approximately 2-µm oval at the base of each flagellum. n > 100 for each condition; one flagellum was measured per cell. ****p < 0.0001 as compared to control (#, top row) or g1 CDKL5-GFP-TG (#, bottom row) cells by one-way ANOVA with Tukey’s multiple comparisons post-hoc test. Violin plots show median (solid line) and quartiles (dashed lines). Underlying data can be found in S1 Data. (TIF) [file pbio.3003560.s005.tif]

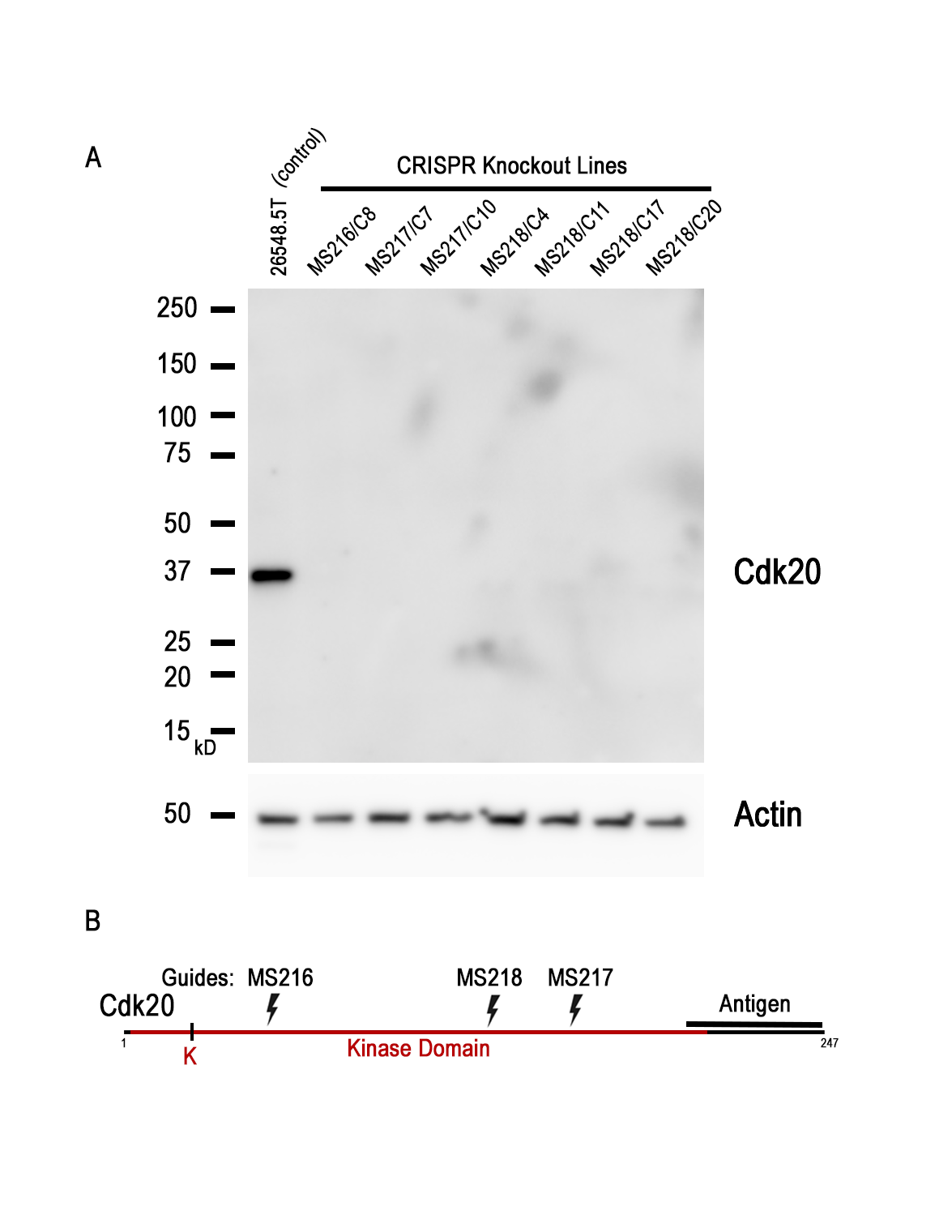

Supplement: S6 Fig — (A) Mouse embryonic fibroblast line 26548.5T was transfected with lentiviral CRISPR guide constructs MS216, MS217, and MS218. After drug selection, the cells were single-cell sorted into 96-well dishes and then screened by western blotting with a Cdk20 antibody using an actin antibody as loading control. (B) Diagram of mouse Cdk20 showing the position of the kinase domain (red), active site lysine (K), positions of the three guides (MS216, MS217, and MS218) and the location of the antigen used to generate the antibody used. (TIF) [file pbio.3003560.s006.tif]

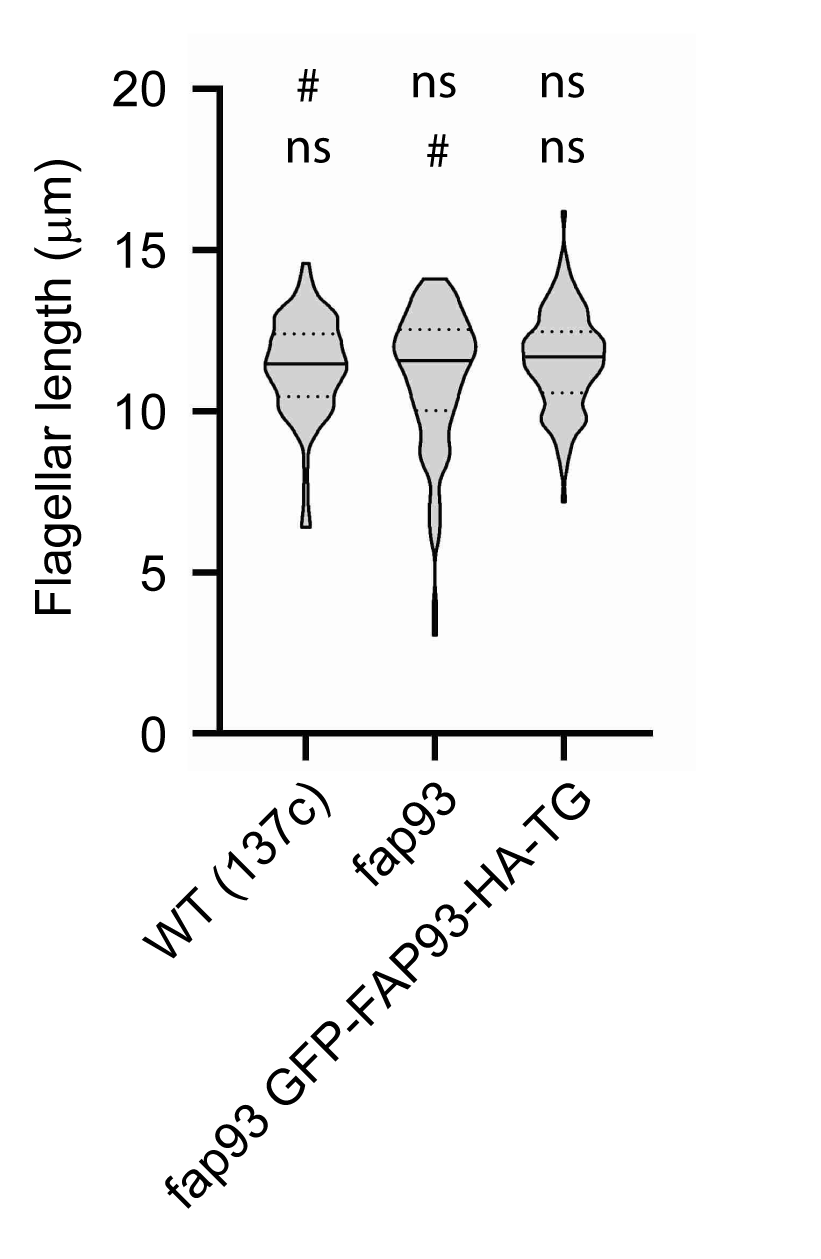

Supplement: S7 Fig — Flagella lengths of wild-type cells (137c), fap93 cells, and fap93 GFP-FAP93-HA-TG cells are shown. One flagellum from each of 50 cells was measured for each cell line. No significant difference (ns) was identified among the three strains by one-way ANOVA with Tukey’s multiple comparisons post-hoc test. Top row shows the pairwise comparison to wild-type cells (#) and bottom row shows the pairwise comparison to fap93 cells (#). Violin plots show median (solid line) and quartiles (dashed lines). Underlying data can be found in S1 Data. (TIF) [file pbio.3003560.s007.tif]

# Figure 4

B

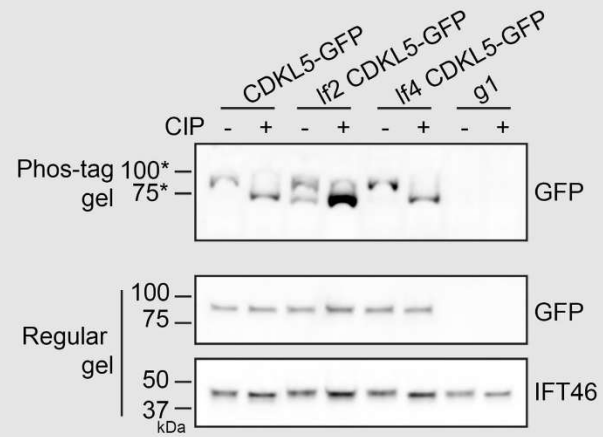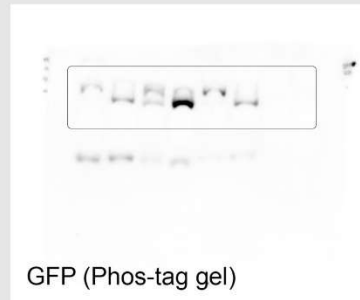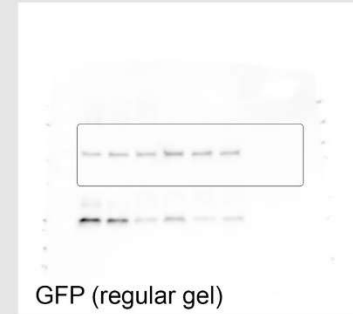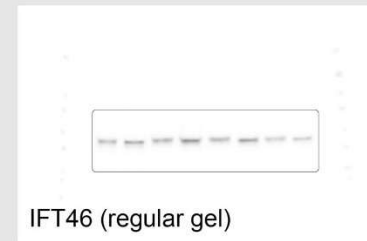

C

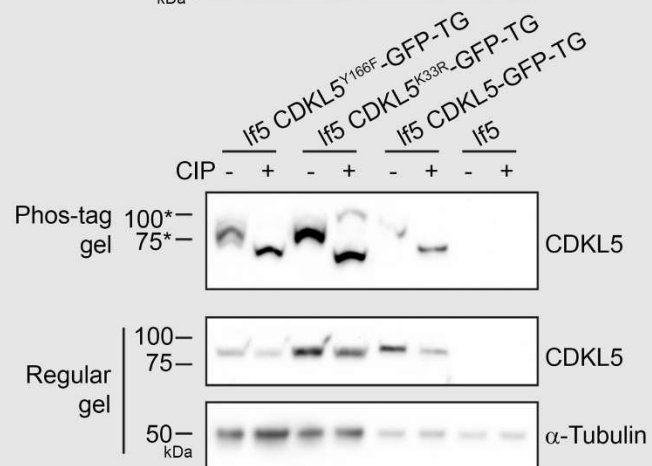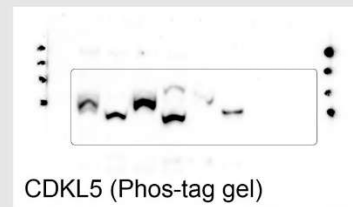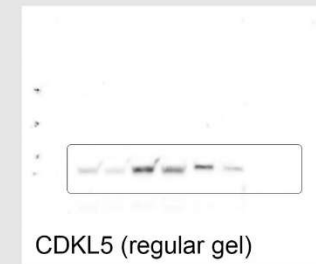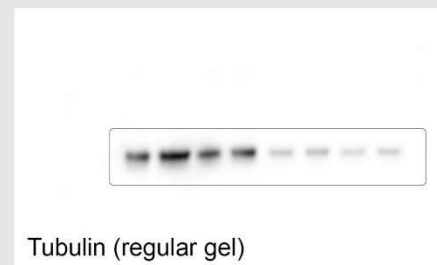

# Figure 6

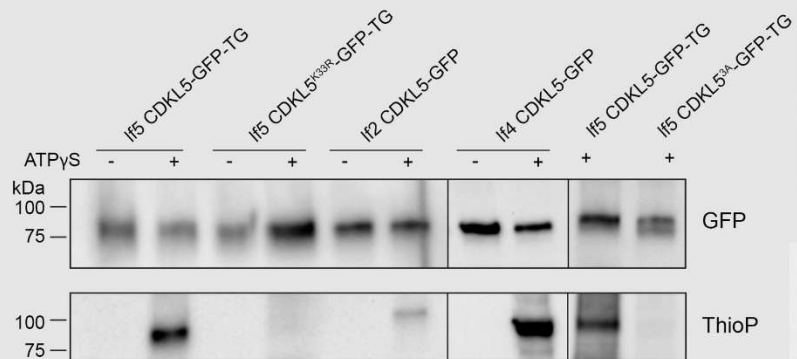

Lanes 1-6

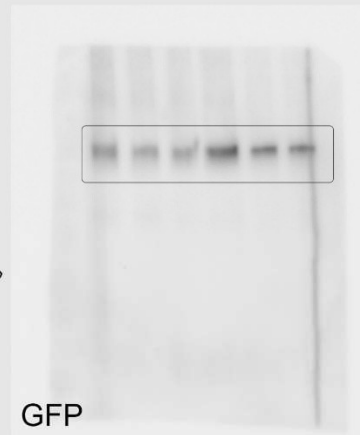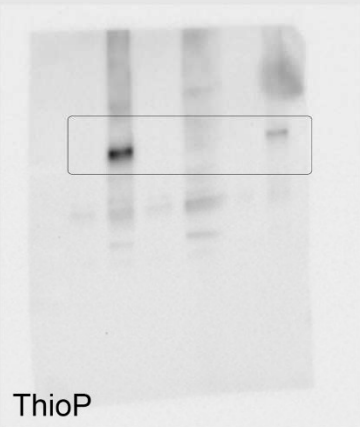

Lanes 7-8

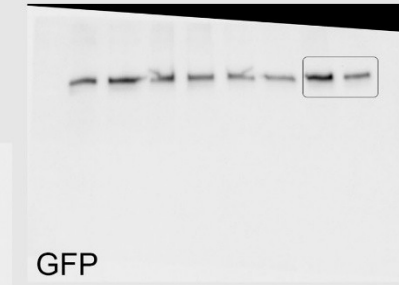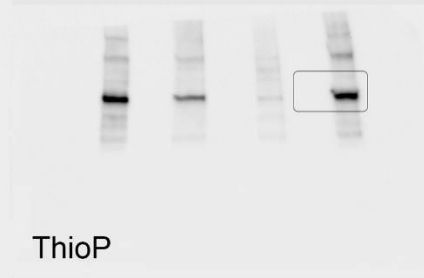

Lanes 9-10

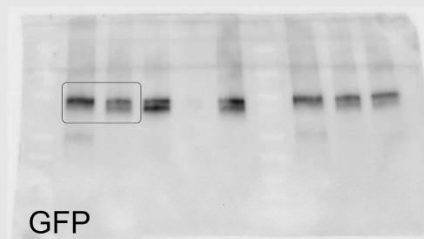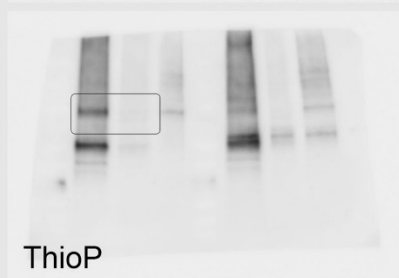

# Figure 7A

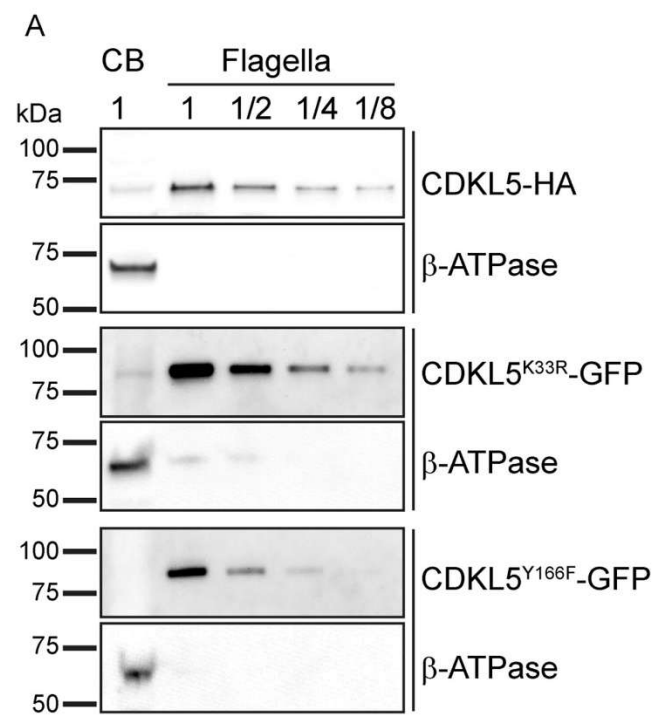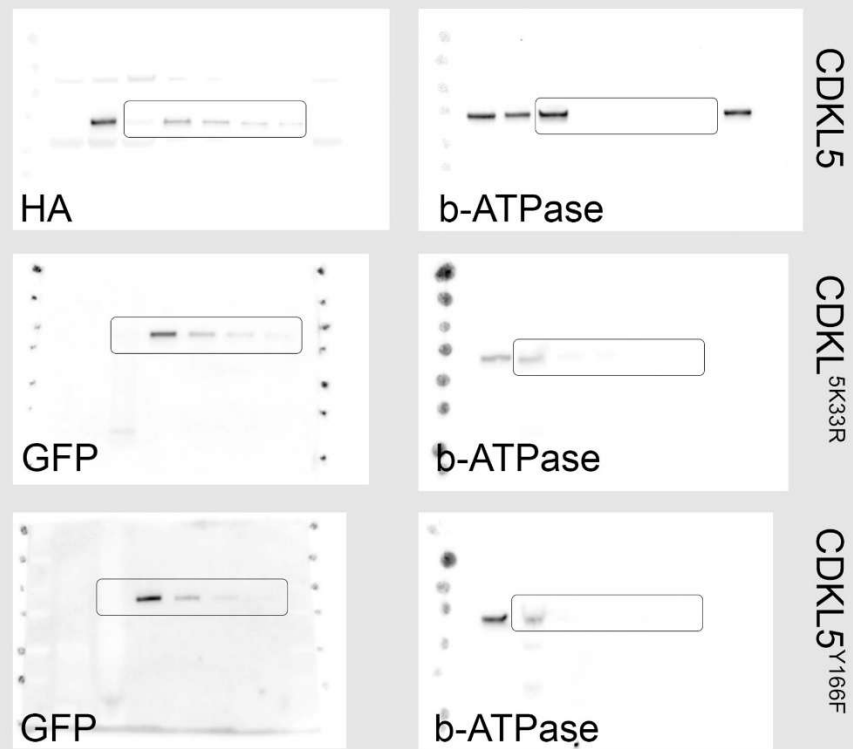

# Figure 7B

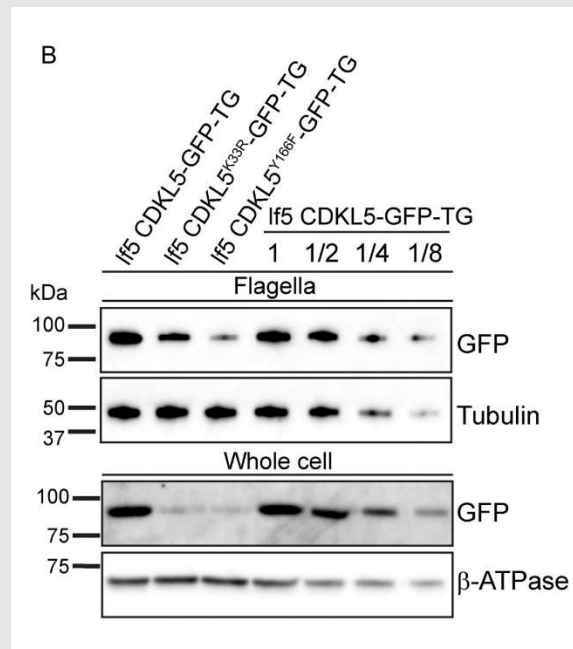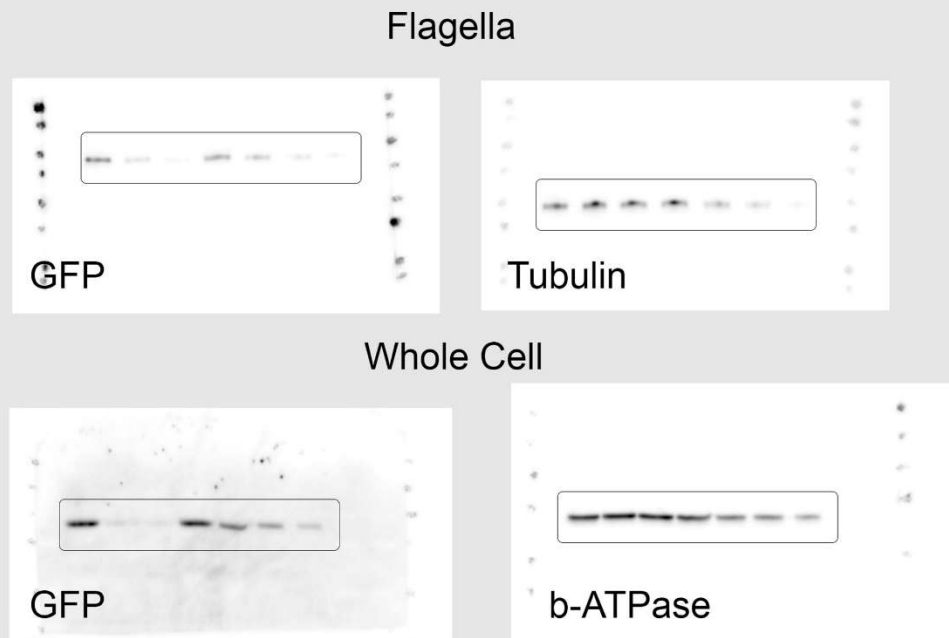

# S2 Figure

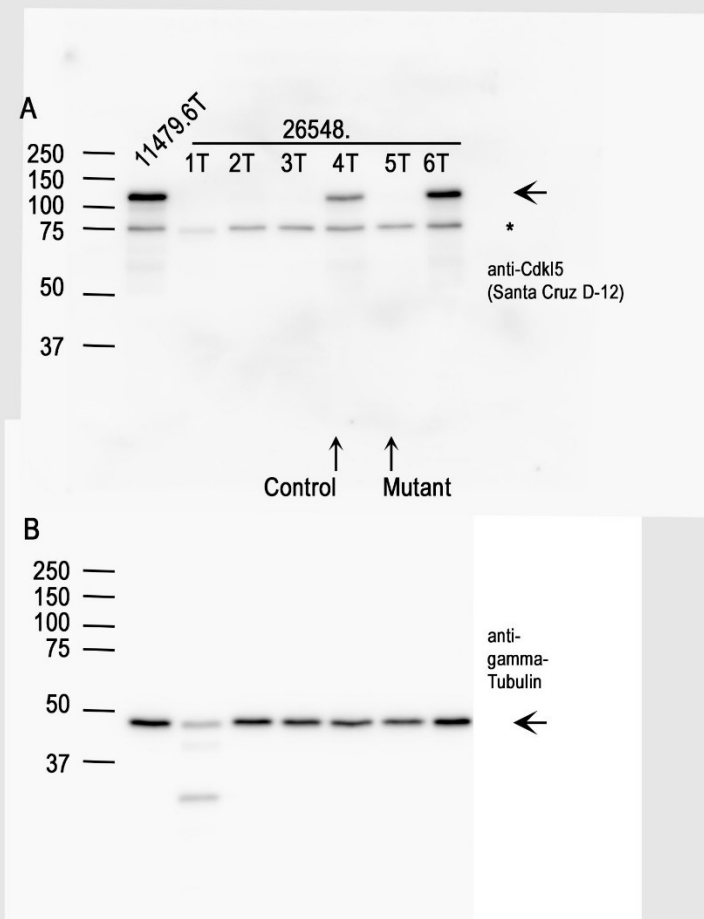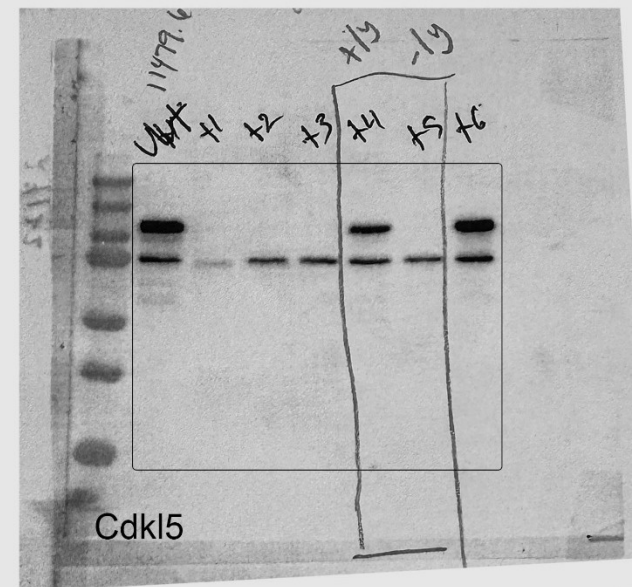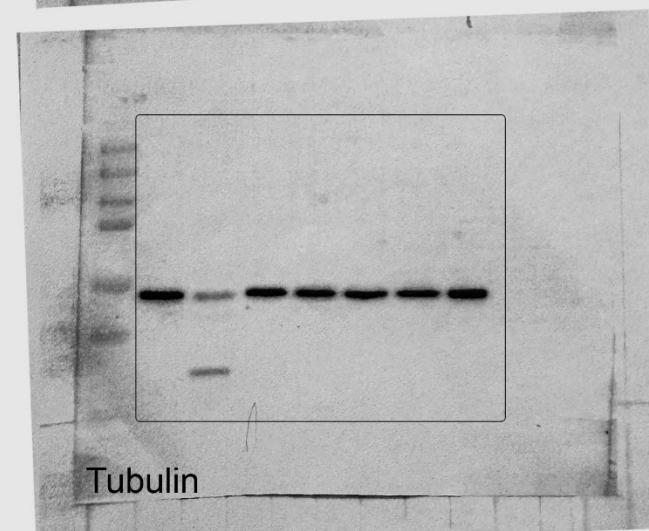

# S3 Figure

A

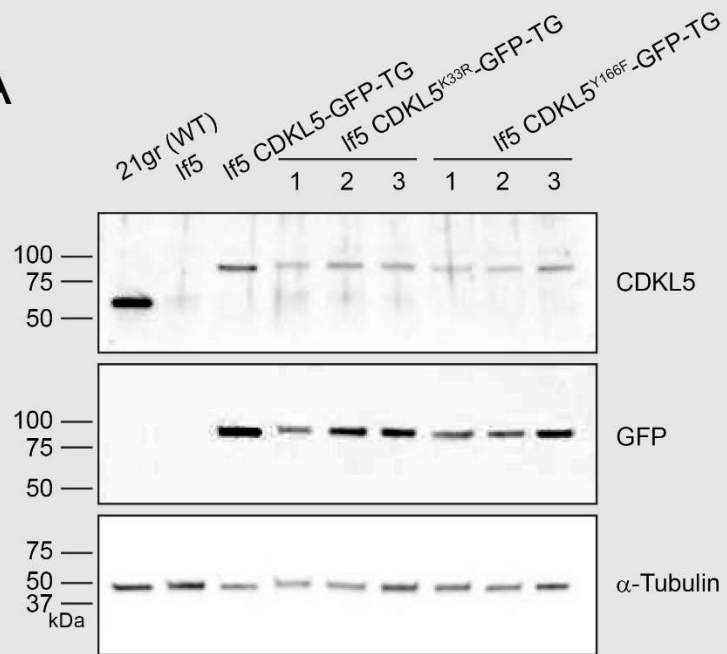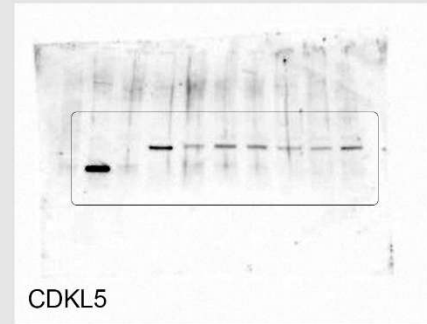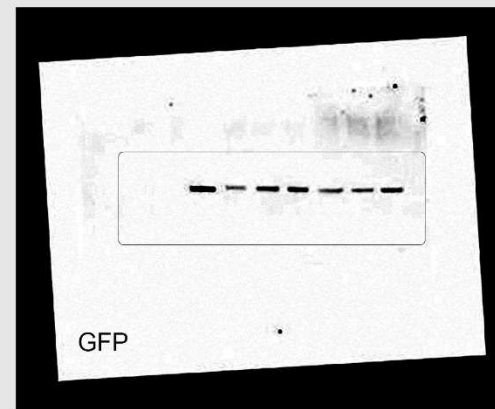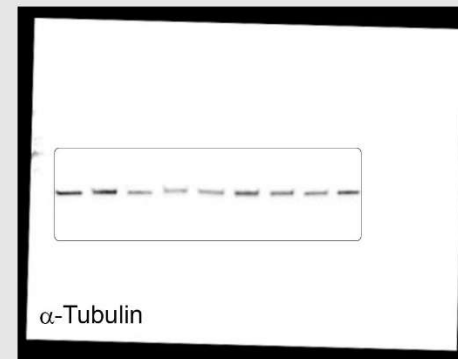

# S4 Figure

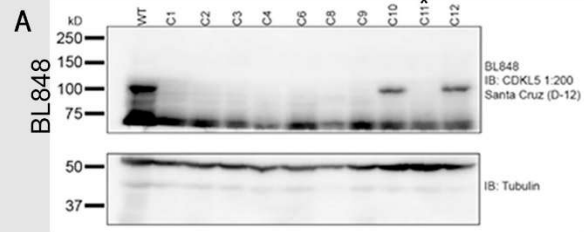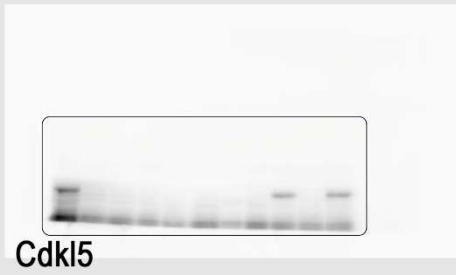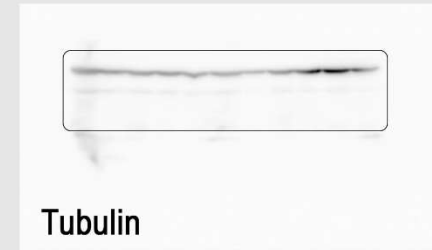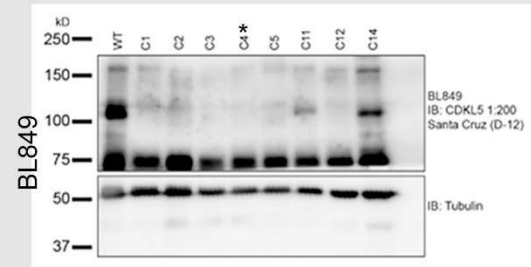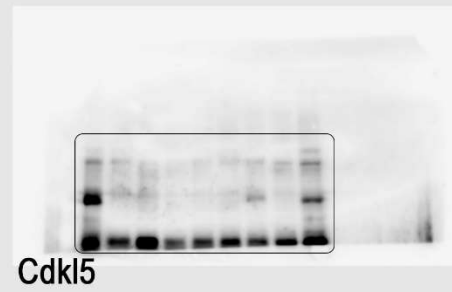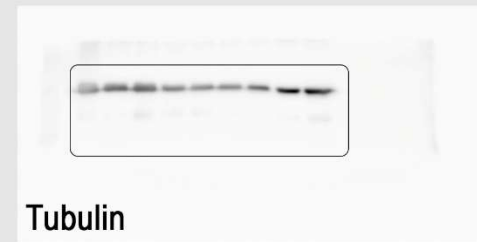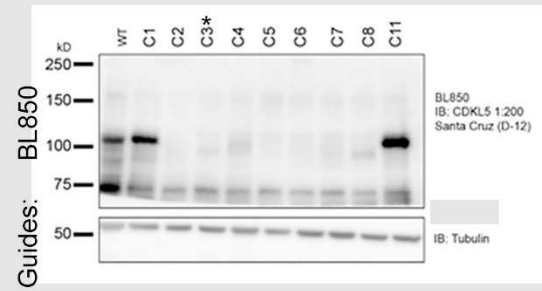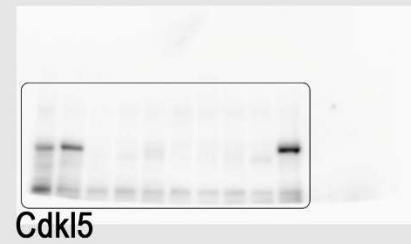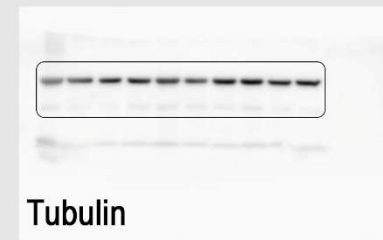

# S6 Figure

A

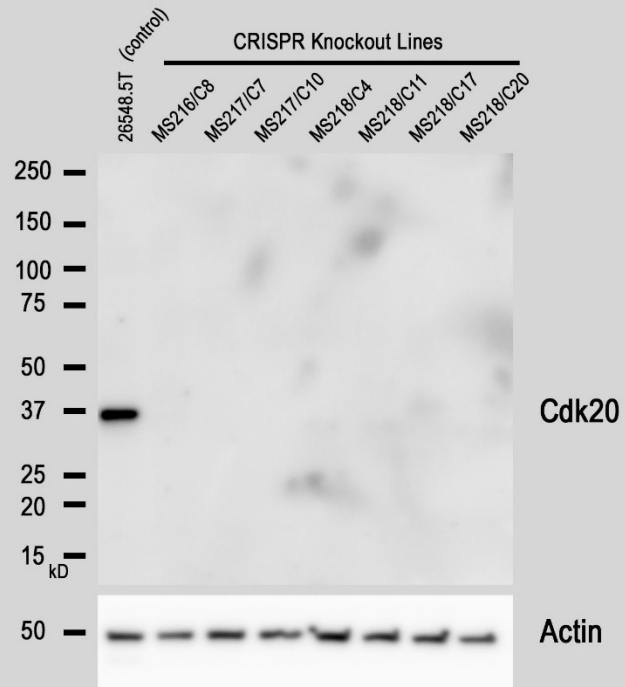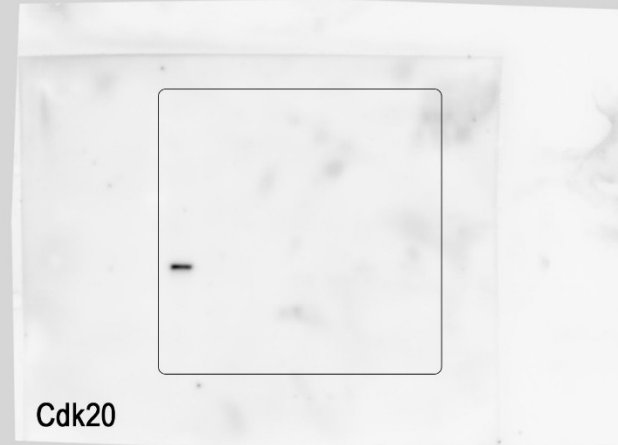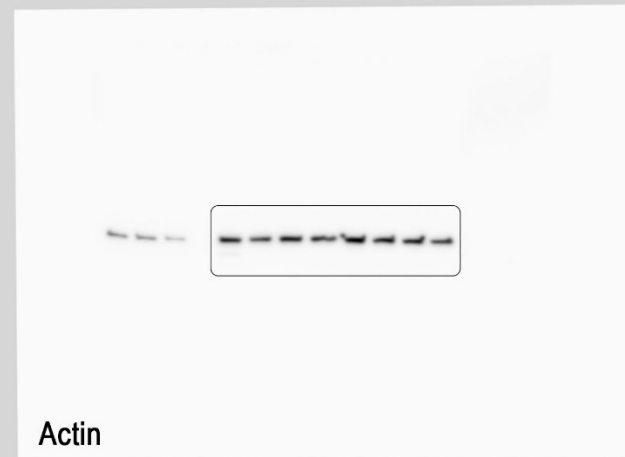

Supplement: S1 Raw Images — (PDF) [file pbio.3003560.s015.pdf]
