# Supplementary material for: Activation of the ciliary kinase CDKL5 is mediated by the cyclin-dependent kinase CDK20/LF2 to control flagellar length
Source: PLoS Biol. 2025 Dec 12;23(12):e3003560. doi: 10.1371/journal.pbio.3003560 (PMC12711092; doi:10.1371/journal.pbio.3003560)
Supplement: S2 Data — Schematic of Chlamydomonas chromosome 12 surrounding the CDKL5 locus. Scale bar is 1 kb. Positions of the PCR products are shown in black if the region was not deleted in lf5-2 and fuchsia if it was deleted. Genomic DNA from wild-type (21gr, CC-1690) and lf5-2 (CC-4560) was amplified with GoTaq (M7122, Promega Corp, Madison, WI, USA) using the primers listed below. In each gel, lane 1 is wild-type, lane 2 is lf5-2 and lane 3 is a no DNA added control. Ladder is 1 kb extended DNA ladder (N3239, NEB, Ipswich, MA, USA). In addition to CDKL5, Cre12.g538250, and Cre12.g538200 were also deleted. Cre12.g538250 is defined at JGI as sucrose 6-glucosyltransferase and is only conserved within Chlamydomonadales. Cre12.g538200 is defined at JGI as 50S ribosome-binding GTPase (MMR_HSR1)//Serine hydrolase (FSH1) (FSH1)//RWD domain (RWD)//Obg-like GTPase YGR210-like, G4 motif-containing domain (YGR210-like_G4) and is conserved in algae and invertebrates. Neither gene has been connected to flagella in any study. (DOCX) [file pbio.3003560.s009.docx]

**S2 Data**


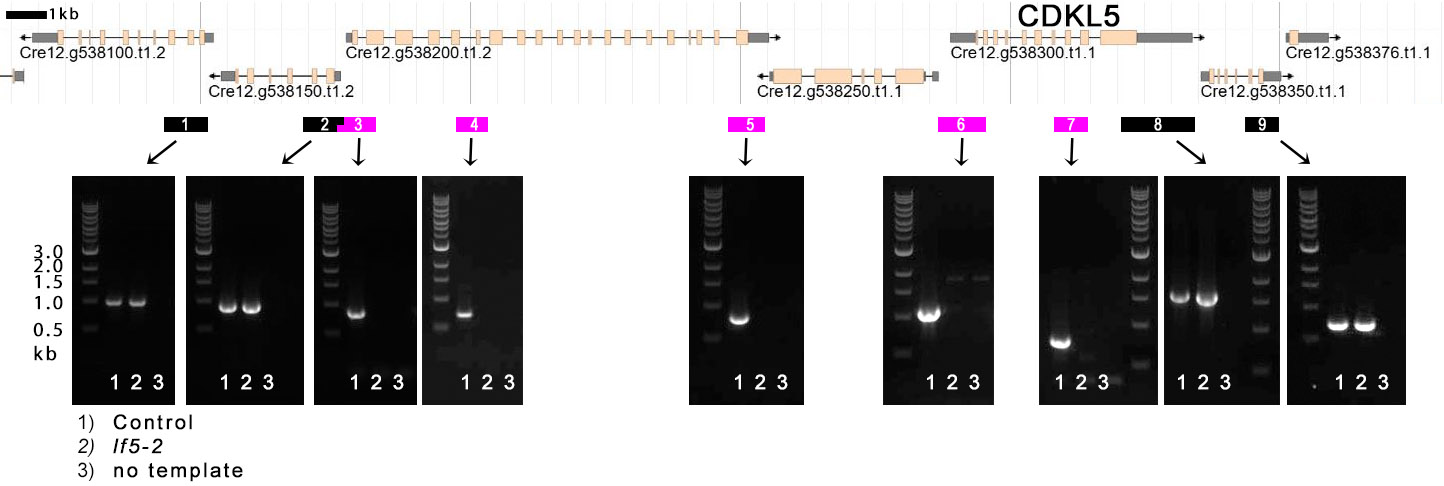


Schematic of *Chlamydomonas* chromosome 12 surrounding the CDKL5 locus. Scale bar is 1 kb. Genomic DNA from wild type (21gr, CC-1690) and lf5-2 (CC-4560) was amplified with Gotaq (M7122, Promega Corp, Madison WI USA) using the primers listed below. In each gel, lane 1 is wild type, lane 2 is *lf5-2* and lane 3 is a no-DNA-added control. Ladder is 1kb extended DNA ladder (N3239, NEB, Ipswich MA USA). Positions of the PCR products are shown in black if the region was not deleted in *lf5-2* and fuchsia if it was deleted.

In addition to most of CDKL5, Cre12.g538250 and Cre12.g538200 were also deleted. Cre12.g538250 is defined at JGI as sucrose 6-glucosyltransferase and is only conserved within *Chlamydomonadales*. Cre12.g538200 is defined at JGI as 50S ribosome-binding GTPase (MMR_HSR1) // Serine hydrolase (FSH1) (FSH1) // RWD domain (RWD) // Obg-like GTPase YGR210-like, G4 motif-containing domain (YGR210-like_G4) and is conserved in algae and invertebrates. Neither gene has been connected to flagella in any study.

| PCR product number | Primer name | Primer sequence | Product size (bp) | Result |
| --- | --- | --- | --- | --- |
| 1 | LF5-50 | TTCGCCTTTACAACCAACCG | 993 | normal |
|  | LF5-59 | TTGCTTTGGGTTCCGGAATG |  |  |
| 2 | LF5-52 | GAACCATCCCCATTCGCTTC | 914 | normal |
|  | LF5-61 | ACCACGTGATATCCGGTACC |  |  |
| 3 | LF5-54 | CCGACCCGTTCTGCTTTAAG | 866 | deleted |
|  | LF5-63 | CAGATCGAAGCGGGTGAAAG |  |  |
| 4 | LF5-44 | CGCTCCGTTGAACTCTCTTG | 710 | deleted |
|  | LF5-53 | TCCCTCACCTTCACGTTACC |  |  |
| 5 | LF5-46 | CACTCTGCCCTGTAATGTGC | 821 | deleted |
|  | LF5-55 | CTTTGCAACCCCTACCCAAC |  |  |
| 6 | LF5-23 | AGGTTTTGCCACCTTGATTG | 1141 | deleted |
|  | LF5-14 | CACCGCTACAATCTCTCCCG |  |  |
| 7 | LF5-13 | ACATACTGCAGCGGTTGC | 784 | deleted |
|  | LF5-12 | GGGCATGTCCTCGTCCAT |  |  |
| 8 | LF5-37 | CACGAGTATCGCGTCGTCTG | 1677 | normal |
|  | LF5-30 | GGCTCGAACTCCTAGTCAACG |  |  |
| 9 | LF5-38 | TGTACTCTGGGGCTGACATG | 738 | normal |
|  | LF5-47 | TCAGTAAGCTCTCTCGCCTG |  |  |
