# Supplementary material for: Activation of the ciliary kinase CDKL5 is mediated by the cyclin-dependent kinase CDK20/LF2 to control flagellar length
Source: PLoS Biol. 2025 Dec 12;23(12):e3003560. doi: 10.1371/journal.pbio.3003560 (PMC12711092; doi:10.1371/journal.pbio.3003560)
Supplement: S1 Table — Primers used for amplification of Chlamydomonas sequences. (DOCX) [file pbio.3003560.s016.docx]

#### S1 Table. *Chlamydomonas* primers

| Name | Sequence 5’-3’ | Description |
| --- | --- | --- |
| LF5donor-1 | AGCCGGTGCTGTACCAGACCAATGCCGCTGCGGGCGCCAGTAAG | Amplification of donor from pLF5CsfGFP plasmid for LF5 C-terminus TIM tagging |
| LF5donor-2 | GCTGGTACCATCAACTGACGTTAC |  |
| LF5donor-1 | AGCCGGTGCTGTACCAGACCAATGCCGCTGCGGGCGCCAGTAAG | Amplifies donor from pLF5CsfGFPHyg for LF5 C-terminus TIM tagging |
| Hyg-4 | CCCGGTACCCGCTTCAAATA |  |
| LF5-41 | ATGATTACGAATTCGATGCACGAGCCCATGGACCA | Amplifies product from pLF5CsfGFP to construct pLF5CsfGFPHyg plasmid |
| LF5-34 | GCAAGAAAGAAGCTTGATAGTAAGGAGGCTCGAACTCC |  |
| LF5-37 | CACGAGTATCGCGTCGTCTG | PCR screening for LF5 C-terminus sfGFP tagging |
| sfGFP-4 | GAACTTCAGGGTCAGCTTGC |  |
| LF5-25 | CGTGTCCACTCGCTGGTAC | Amplifies product for pLF5-K33R, pLF5-Y166F, and pLF5-Y166A construction |
| LF5-14 | CACCGCTACAATCTCTCCCG |  |
| LF5-16 | CGGTACCAGCGAGTGGACACG | Amplifies product for pLF5-K33R construction |
| LF5-27 | GAGAGATTGTAGCGGTGcgcAAGTTTAAAGAAAGCGACG |  |
| LF5-18 | CCAGCGAGTGGACACGaAGTCAGTGATGGATACGT | Amplifies product for pLF5-Y166F construction |
| LF5-29 | GGAAACGGGAGAGATTGTAGCGGTG |  |
| LF5-29 | GGAAACGGGAGAGATTGTAGCGGTG | Amplifies product for pLF5-Y166A construction |
| LF5-22 | CCAGCGAGTGGACACGgcGTCAGTGATGGATACGTC |  |
| LF5-14 | CACCGCTACAATCTCTCCCG | Amplifies product for pLF5-S162,T164,Y166 construction |
| LF5-45 | CTGACgcCGTGTCCACTC |  |
| LF5-29 | GGAAACGGGAGAGATTGTAGCGGTG | Amplifies product for pLF5-S162,T164,Y166 construction |
| LF5-36 | GTGGACACGgcGTCAGcGATGGcTACGTCAGCGGGAGGCAG |  |
| Fus-3 | TCCAACGCATAGCCATCAAC | Mating type plus specific gene as PCR control |
| Fus-4 | TGTTTGCTAGGGGTGCAATG |  |
| Mid-1 | ACCGGTGTTTACCGTCGAGT | Mating type minus specific gene as PCR control |
| Mid-2 | CCTTTCTGTAGGGCCACCTG |  |
