# Supplementary material for: Activation of the ciliary kinase CDKL5 is mediated by the cyclin-dependent kinase CDK20/LF2 to control flagellar length
Source: PLoS Biol. 2025 Dec 12;23(12):e3003560. doi: 10.1371/journal.pbio.3003560 (PMC12711092; doi:10.1371/journal.pbio.3003560)
Supplement: S2 Table — Antibodies used in this work. (DOCX) [file pbio.3003560.s017.docx]

#### S2 Table. Antibodies

| **Antibody (Antigen Species)** | **Species Raised In** | **Clone or Antibody Name (Isotype)** | **Catalog Number, Supplier** | **Dilution** |
| --- | --- | --- | --- | --- |
| **Primaries** |  |  |  |  |
| CDKL5 (human) | Mouse | D12 (IgG2b) | sc-376314, Santa Cruz Biotech, Houston TX USA | 1:200-1:1000 |
| CDKL5 (*Chlamydomonas*) | Rabbit |  | Tam et al. 2013. PMID: 23283985 | 1:5000 |
| CDK20 (human) | Rabbit |  | PA5-55593, Invitrogen, Waltham MA USA | 1:1000 |
| Myc | Mouse | 9E10 | sc-40, Santa Cruz Biotechnology, Houston TX USA | 1:500 |
| HA | Rat | 3F10 | 11867423001, Sigma-Aldrich, St. Louis MO USA | 1:2000 |
| CEP164 (human) | Rabbit |  | 22227-1-AP, Proteintech, Rosemont IL USA | 1:1000 |
| Arl13b (mouse) | Mouse | N295B/66 (IgG2a) | NIH NeuroMab, Davis CA USA | 1:1000 |
| γ-Tubulin (human) | Mouse | GTU-88 (IgG1) | T5326, Sigma-Aldrich, St. Louis MO USA | 1:10K |
| α-Tubulin (sea urchin) | Mouse | B-5-1-2 (IgG1) | T5168, Sigma-Aldrich, St. Louis MO USA | 1:5000 |
| GAPDH (human) | Rabbit | 14C10 | 3683S, Cell Signaling, Danvers MA USA | 1:5000 |
| Acetylated α-tubulin (sea urchin) | Mouse | 6-11B-1 (IgG2b) | T7451, Sigma-Aldrich, St. Louis MO USA | 1:1000-  1:10000 |
| GFP | Mouse | 13.1+7.1 (IgG1κ) | 11814460001, Roche, Basal Switzerland | 1:100 for IF; 1:1000 for WB |
| GFP | Rabbit | (IgG) | G10362, Thermo Fisher, Waltham MA USA | 1:500 |
| GFP | Mouse | JL-8 | 632381, Clontech, Mountain View CA USA | 1:1000 |
| IFT46 (*Chlamydomonas*) | Guinea Pig |  | Hou and Witman 2017. PMID: 28701346 | 1:10K |
| ATP synthase β subunit (*Chlamydomonas*) | Rabbit |  | AS05 085, Agrisera, Vännäs Sweden | 1:100K |
| Thiophosphate ester | Rabbit | 51-8 (IgG) | ab92570, Abcam, Cambridge UK | 1:6000 |
| **Secondaries** |  |  |  |  |
| Rabbit IgG-Alexa 488 | Goat |  | A11034, Invitrogen, Waltham MA USA | 1:1000 |
| Rabbit IgG-Alexa 568 | Goat |  | A11011, Invitrogen, Waltham MA USA | 1:1000 |
| Rabbit IgG-Alexa 594 | Donkey |  | A21207, Invitrogen, Waltham MA USA | 1:1000 |
| Goat IgG-Alexa 594 | Donkey |  | A11058, Invitrogen, Waltham MA USA | 1:1000 |
| Mouse IgG-Alexa 488 | Goat |  | A11017, Invitrogen, Waltham MA USA | 1:1000 |
| Mouse IgG-Alexa 488 | Donkey |  | A32766, Invitrogen, Waltham MA USA | 1:1000 |
| Mouse IgG-Alexa 568 | Donkey |  | A10037, Invitrogen, Waltham MA USA | 1:1000 |
| Mouse IgG-Alexa 594 | Goat |  | A11032, Invitrogen, Waltham MA USA | 1:1000 |
| Mouse IgG1-Alexa 488 | Goat |  | A21121, Invitrogen, Waltham MA USA | 1:1000 |
| Mouse IgG1-Alexa 568 | Goat |  | A21124, Invitrogen, Waltham MA USA | 1:1000 |
| Mouse IgG2a-Alexa 488 | Goat |  | A21131, Invitrogen, Waltham MA USA | 1:1000 |
| Mouse IgG2a-Alexa 568 | Goat |  | A21134, Invitrogen, Waltham MA USA | 1:1000 |
| Mouse IgG2b-Alexa 488 | Goat |  | A21141, Invitrogen, Waltham MA USA | 1:1000 |
| Mouse IgG2b-Alexa 568 | Goat |  | A21144, Invitrogen, Waltham MA USA | 1:1000 |
| Rabbit IgG-HRP | Goat |  | G-21234, Invitrogen, Carlsbad CA USA | 1:2000 |
| Guinea Pig IgG-HRP | Rabbit |  | A5545, Sigma-Aldrich, St. Louis MO USA | 1:2000 |
| Mouse IgG-HRP | Goat |  | A4416, Sigma-Aldrich, St. Louis MO USA | 1:2000 |
| Rat IgG-HRP | Goat |  | 112-036-003, Jackson Immuno Research, West Grove PA USA | 1:10K |
