# Supplementary material for: Activation of the ciliary kinase CDKL5 is mediated by the cyclin-dependent kinase CDK20/LF2 to control flagellar length
Source: PLoS Biol. 2025 Dec 12;23(12):e3003560. doi: 10.1371/journal.pbio.3003560 (PMC12711092; doi:10.1371/journal.pbio.3003560)
Supplement: S3 Table — Plasmids created in this study. (DOCX) [file pbio.3003560.s018.docx]

#### S3 Table. Plasmids

| Name | Description | Insert | Tags | Selection |
| --- | --- | --- | --- | --- |
| MS234 | Cdkl5-6xMyc | *M. musculus* Cdkl5 | 6xMyc | Bsd |
| MS236 | Cdkl5-6xMyc (CRISPR resistant) | *M. musculus* Cdkl5 | 6xMyc | Bsd |
| MS238 | Cdkl5^K42A^-6xMyc | *M. musculus* Cdkl5 mutant | 6xMyc | Bsd |
| GP1192 | Cdkl5^T169A,Y171F^-6xMyc | *M. musculus* Cdkl5 mutant | 6xMyc | Bsd |
| BL848 | Cdkl5 guide1 | ACATAGAAACCAATCCACCA |  | Puro |
| BL849 | Cdkl5 guide1 | AAGAACGACATCGTCCACAG |  | Puro |
| BL850 | Cdkl5 guide1 | TCGGAGAGGAAAATTATACC |  | Puro |
| MS216 | Cdk20 guide 1 | GCACAAAGCCCGCACCATGT |  | Puro |
| MS217 | Cdk20 guide 2 | CGGCCAGACTCGAGGACTCG |  | Puro |
| MS218 | Cdk20 guide 3 | CTCACCATAGGTCAACGCCC |  | Puro |
| pLF5CsfGFPHyg | 3’ end CDKL5-GFP | *Chlamydomonas* CDKL5 3’ end | sfGFP | Hyg |
| pLF5CsfGFP | CDKL5-GFP | *Chlamydomonas* CDKL5 | sfGFP | Paro |
| pLF5-K33R | CDKL5^K33R^-GFP | *Chlamydomonas* CDKL5 mutant | sfGFP | Paro |
| pLF5-Y166F | CDKL5^Y166F^-GFP | *Chlamydomonas* CDKL5 mutant | sfGFP | Paro |
| pLF5-S162A,T164A,Y166A | CDKL5^S162A,T164A,Y166A^-GFP | *Chlamydomonas* CDKL5 mutant | sfGFP | Paro |
