# Supplementary material for: Activation of the ciliary kinase CDKL5 is mediated by the cyclin-dependent kinase CDK20/LF2 to control flagellar length
Source: PLoS Biol. 2025 Dec 12;23(12):e3003560. doi: 10.1371/journal.pbio.3003560 (PMC12711092; doi:10.1371/journal.pbio.3003560)
Supplement: S4 Table — Overview of all of the MS runs analyzed in this study. (DOCX) [file pbio.3003560.s019.docx]

#### S4 Table. MS experiments

| **MS experiment** | **Goal** | **Biological sample** | **MS quantification method** | **MS Facility** | **ProteomeXchange (https://www.ebi.ac.uk/pride/login)**  **Database**  **Accession Number** |
| --- | --- | --- | --- | --- | --- |
| Experiment 1 | CDKL5 phosphorylation state analysis | CDKL5-GFP purified from lf5 CDKL5-GFP-TG whole cell lysate | NA | Targeted Protein Degradation Proteomics Core at the Dana-Farber Cancer Institute | **Project accession:** PXD066796  **Reviewer Token:** 6iRjW0rhspys |
| Experiment 2 | CDKL5 phosphorylation state analysis | CDKL5-GFP purified from lf5 CDKL5-GFP-TG whole cell lysate | NA | Targeted Protein Degradation Proteomics Core at the Dana-Farber Cancer Institute | **Project accession:** PXD066796  **Reviewer Token:** 6iRjW0rhspys |
| Experiment 3 | CDKL5 phosphorylation state analysis | CDKL5-GFP purified from lf5 CDKL5-GFP-TG whole cell lysate, CDKL5-GFP from lf2 CDKL5-GFP whole cell lysate, and CDKL5^K33R^-GFP purified from lf5 CDKL5^K33R^-GFP-TG whole cell lysate | Label free | Mass Spectrometry Facility at UMass Chan Medical School | **Project accession:** PXD068782  **Reviewer Token:** ceFkcezUGdfn |
| Experiment 4 | CDKL5 phosphorylation state analysis | CDKL5-GFP purified from lf5 CDKL5-GFP-TG whole cell lysate, CDKL5-GFP from lf2 CDKL5-GFP whole cell lysate, and CDKL5^K33R^-GFP purified from lf5 CDKL5^K33R^-GFP-TG whole cell lysate | Label free | Mass Spectrometry Facility at UMass Chan Medical School | **Project accession:** PXD068782  **Reviewer Token:** ceFkcezUGdfn |
| Experiment 5 | CDKL5 phosphorylation state analysis | CDKL5-GFP purified from lf5 CDKL5-GFP-TG whole cell lysate, treated with and without CIP | Label free | Mass Spectrometry Facility at UMass Chan Medical School | **Project accession:** PXD068782  **Reviewer Token:** ceFkcezUGdfn |
| Experiment 6 | CDKL5 phosphorylation state analysis | CDKL5-GFP purified from lf5 CDKL5-GFP-TG whole cell lysate, treated with and without CIP | Label free | Mass Spectrometry Facility at UMass Chan Medical School | **Project accession:** PXD068782R  **Reviewer Token:** ceFkcezUGdfn |
| Experiment 7 | CDKL5 phosphorylation state analysis | CDKL5^K33R^-GFP purified from lf5 CDKL5^K33R^-GFP-TG whole cell lysate, treated with and without CIP | Label free | Mass Spectrometry Facility at UMass Chan Medical School | **Project accession:** PXD068782  **Reviewer Token:** ceFkcezUGdfn |
| Experiment 8 | Proteome and phosphoproteome analysis | Purified flagella from lf5 and 21gr | TMT labelling | Vermont Biomedical Research Network’s Proteomics Facility at the University of Vermont | **Project accession:** PXD066877  **Reviewer Token:** UG2dGZfsqJqR |
| Experiment 9 | Proteome and phosphoproteome analysis | Purified flagella from lf5 and 21gr | TMT labelling | Vermont Biomedical Research Network’s Proteomics Facility at the University of Vermont | **Project accession:** PXD066877  **Reviewer Token:** UG2dGZfsqJqR |
| Experiment 10 | Identification of CDKL5-interacting proteins | Immunoprecipitation products from lf5 CDKL5-GFP-TG and lf5 whole cell lysate | Label free | Mass Spectrometry Facility at UMass Chan Medical School | **Project accession:** PXD068782  **Reviewer Token:** ceFkcezUGdfn |
| Experiment 11 | Identification of CDKL5-interacting proteins | Immunoprecipitation products from lf5 CDKL5-GFP-TG and lf5 whole cell lysate | Label free | Mass Spectrometry Facility at UMass Chan Medical School | **Project accession:** PXD068782  **Reviewer Token:** ceFkcezUGdfn |
