# Supplementary material for: Activation of the ciliary kinase CDKL5 is mediated by the cyclin-dependent kinase CDK20/LF2 to control flagellar length
Source: PLoS Biol. 2025 Dec 12;23(12):e3003560. doi: 10.1371/journal.pbio.3003560 (PMC12711092; doi:10.1371/journal.pbio.3003560)
Supplement: S1 Text — Detailed methods for MS analysis. (DOCX) [file pbio.3003560.s020.docx]

S1 Text

# Abbreviations:

FDR: False Discovery Rate

# Materials and Methods Supplementary File

## Mass spectrometry analysis

### Biological sample preparation for analysis of phosphorylation states of CDKL5 for Experiments 1 to 7

For Experiments 1 to 4, GFP-tagged CDKL5 proteins were purified from soluble cell lysates. Soluble cell lysates were made by first harvesting cells from one to four liters of mid-log phase culture [57]. Cells were washed twice with 10 mM HEPES pH 7.4. The cells were then resuspended in 10 mM HEPES pH 7.4 in a minimum volume. Protease and phosphatase inhibitors (A32961, Pierce Protease and Phosphatase Inhibitor Mini Tablets, Thermo Scientific, Waltham MA USA), which were dissolved in 1 ml distilled water shortly before use, were added to give the final dilution recommended by the manufacturer. NaCl and NP-40 were added to final concentrations of 400 mM and 0.5% respectively; After incubation on ice for 3-5 min, the soluble fraction was separated from the cell body by centrifugation at 16,000 rcf for 10 min at 4°C. Soluble cell lysates were then applied to GFP-affinity beads (gtma, ProteinTech, Rosemont IL USA) equilibrated with wash buffer (10 mM HEPES pH 7.4, 50 mM NaCl, 0.5 mM EDTA, 0.05% NP-40, with protease and phosphatase inhibitors [A32961, Thermo Scientific, Waltham MA USA]) and incubated at 4°C for 4 hr. The beads were then washed four times with wash buffer at room temperature. Finally, 2.5X protein loading buffer (0.025 M Tris-HCl pH 8, 80 mM DTT, 2.5 mM EDTA pH 8, 25% sucrose, 2.5% SDS, 0.02% [w/v] bromophenol blue) was added to the beads and boiled for 8 min to elute the proteins. The eluates were then separated by SDS-PAGE, stained with Coomassie blue, and the bands corresponding to the GFP-tagged CDKL5 were excised for analysis by MS in the Targeted Protein Degradation Proteomics Core at the Dana-Farber Cancer Institute and at the Mass Spectrometry Facility at UMass Chan Medical School.

For Experiments 5, 6, and 7, CDKL5-GFP beads were prepared as in MS Experiments 1-4. One half of the beads was kept on ice as a control, while the other half was rinsed three times with wash buffer lacking protease and phosphatase inhibitors. The washed beads were then washed once with 1X rCutSmart buffer (B6004S, New England Biolabs, Ipswich MA USA) and then treated twice with Quick CIP (M0525S, New England Biolabs, Ipswich MA USA) in 1X rCutSmart buffer for 30 min with rotation at 37°C; the beads were washed once with 1X rCutSmart buffer between the two CIP treatments. The control beads and the CIP-treated beads were rinsed with wash buffer twice and eluted as described in the preceding paragraph. The eluates were then separated by SDS-PAGE, stained with Coomassie blue, and the bands corresponding to the GFP-tagged CDKL5 were excised for analysis by MS at the Mass Spectrometry Facility at UMass Chan Medical School.

### Biological sample preparation for proteomic and phosphoproteomic analyses for Experiments 8 and 9

For proteomic and phosphoproteomic analyses, flagella were prepared as described [57]. During flagella preparation, phosphatase inhibitors (25 mM NaF, 10 mM Na_4_P_2_O_7_, 1 mM activated Na_3_VO_4_, and 50 mM β-glycerophosphate) were included in the HMS (10 mM HEPES, pH 7.4, 5 mM MgSO_4_, 4% sucrose) and HMS-25% sucrose solutions (10 mM HEPES, pH 7.4, 5 mM MgSO_4_, 25% sucrose). Flagella pellets were resuspended in HMDEK buffer (30 mM HEPES, pH 7.4, 5 mM MgSO_4_, 1 mM DTT, 0.5 mM EGTA, 25 mM KCl) containing proteinase and phosphatase inhibitors (A32961, Pierce Protease and Phosphatase Inhibitor Mini Tablets, Thermo Scientific, Waltham MA USA). A 100-µg aliquot of purified flagella was loaded onto a 4-15% gradient gel and electrophoresed until the front was about 1.5 cm into the gel. The gel was stained with Coomassie blue, destained, and the region containing proteins was excised for analysis by TMT-based MS at the Vermont Biomedical Research Network’s Proteomics Facility at the University of Vermont.

### Biological sample preparation for identification of CDKL5-interacting proteins for Experiments 10 and 11

CDKL5-GFP protein together with its interacting proteins were immunoprecipitated from soluble cell lysates of lf5 CDKL5-GFP-TG and lf5 as described in the section “Biological sample preparation for analysis of phosphorylation states of CDKL5 for Experiments 1 to 7” with the following three changes: 1. To prepare soluble whole-cell lysates, instead of adding NaCl to make a final concentration of 400 mM NaCl, 2 M KCl was added to make a final concentration of 10 mM KCl. 2. ChromoTek GFP-Trap Magnetic Particles (M-270, ProteinTech, Chicago IL USA) were used instead of ChromoTek GFP-Trap Magnetic Agarose beads. 3. Instead of running a fully resolved SDS-polyacrylamide gel and excising the bands corresponding to CDKL5-GFP for MS analysis, the elutes were either electrophoresed briefly on an SDS-polyacrylamide gel and the region containing proteins was excised for MS analysis, or the proteins were eluted from beads with 2% SDS and sent directly for MS analysis.

### In-gel protein digestion and LC-MS/MS analysis for Experiments 1 and 2

Excised gel pieces were destained with 50% acetonitrile in 25 mM ammonium bicarbonate and dehydrated in 100% acetonitrile. Disulfide bonds were reduced with 20 mM dithiothreitol for 30 min at 42°C and alkylated in 55 mM iodoacetamide for 45 min in the dark at RT. Gel pieces were subjected to two rounds of washing in 25 mM ammonium bicarbonate followed by dehydration with 100% acetonitrile. Proteins were digested in 25 mM ammonium bicarbonate with trypsin (1:50 enzyme:protein) for 16 hr at 32°C. Peptides were extracted in 50% acetonitrile/1% formic acid and desalted over C18 ZipTips (ZTC18S, EMD Millipore, Billerica MA USA).

Digested peptides were analyzed on an Orbitrap Eclipse mass spectrometer coupled to an Ultimate 3000 RSLCnano (Thermo Fisher, Waltham MA USA). Peptides were separated over a 50-cm C18 column (ES903, Thermo Fisher, Waltham MA USA) with a 70-min gradient of 6-30% acetonitrile in 0.1% formic acid and electrosprayed (1.9 kV, 300°C) with an EasySpray ion source. Precursor ion scans (375-1,325 m/z) were obtained in the orbitrap (120,000 resolution, profile). Data dependent MS^2^ scans (n = 2 in 15 s, exclusion duration = 30 s) were acquired in the orbitrap following HCD fragmentation (35% NCE, 0.7 m/z isolation, 30,000 resolution).

Raw data were searched against a forward and reverse database containing the LF5 sequence and common contaminants using SEQUEST, permitting a mass tolerance of ±10 ppm, 2 missed cleavages by trypsin, and the following variable modifications: oxidation of methionine, phosphorylation of serine/threonine/tyrosine, carbamidomethylation of cysteine, acrylamidation of cysteine. Spectra of identified phosphopeptides were manually validated to confirm accurate site localization.

### Protein digestion and LC-MS/MS analysis for Experiments 3 to 7, 10 and 11

Briefly, for samples within gel slices, the slices were washed, reduced with DTT, alkylated with iodoacetamide, and then digested overnight in-gel with trypsin at 37°C. The resulting peptide samples were eluted from the gel using 50 mM ammonium bicarbonate, followed by 0.2% formic acid in 80% acetonitrile. For samples in SDS-elution buffer, an equal volume of lysis buffer (5% SDS, 50 mM triethylammonium bicarbonate) was added to initiate the S-Trap Micro Spin digestion protocol (ProtiFi, Fairport NY USA). The proteins underwent reduction by 10mM Tris(2-carboxyethyl)phosphine hydrochloride and alkylation by 20 mM iodoacetamide. Trifluoroacetic acid was used instead phosphoric acid as acidifier [67]. Following an overnight digestion with trypsin at 37°C, peptides were eluted first by addition of 40 µL of 50 mM triethylammonium bicarbonate and spun at 4000 rpm for 1 min. This was followed by a 1-min spin with 40 µL of 0.2% formic acid and, lastly, 40 µL of 50% acetonitrile. The three elutions were then pooled together, dried, and reconstituted in 20 µL of 0.1% formic acid in 5% acetonitrile. After reconstitution, the samples were centrifuged at 16,000 rpm for 16 min, and 18-µL aliquots of the supernatants were transferred to non-binding HPLC vials.

Samples were analyzed using a TimsTOF Pro2 (Bruker, Billerica MA USA) mass spectrometer, which was coupled to a nanoElute LC system (Bruker, Billerica MA USA). For Experiments 3 and 4, each sample was analyzed once. For Experiment 5, each sample was analyzed 3 times, with 1 µl, 3 µl, and 10 µl loading amounts, as technical replicates. For Experiment 6, each sample was analyzed 4 times with 3 µl loading amounts as technical replicates. For Experiment 7, each sample was analyzed 3 times with 3 µl loading amounts as technical replicates. For Experiment 10, each sample was analyzed 4 times as technical replicates, the first three times with 1 µl as the loading amount and the fourth time with 10 µl as the loading amount. For Experiment 11, each sample was analyzed 3 times as technical replicates, with 3 µl as the loading amount each time. The peptides were resolved on a custom-made 25-cm analytical column with a 75-μm inner diameter, packed with ReproSil-Pur C18-AQ, 120 Å, 3 μm (Dr. Maisch GmbH, Ammerbuch-Entringen Germany). The mobile-phase solutions consisted of 0.1% formic acid in water (mobile-phase A) and 0.1% formic acid in acetonitrile (mobile-phase B). A 60-min gradient, gradually increasing mobile-phase B from 5% to 40%, was used. The flow rate was set at 500 nL/min for data-dependent acquisition with parallel accumulation serial fragmentation (DDA-PASEF). The nano-electrospray voltage was kept at 1600V, and the system was configured with a single column method (without a trap).

The DDA-PASEF method involves 10 MS/MS PASEF scans per topN acquisition cycle, with ramp and accumulation times set to 100 ms each. The method covers a mass-to-charge (m/z) range from 100 to 1700 and an ion mobility range (1/K0) from 0.70 to 1.30 V s/cm². Collision energy settings follow a linear function based on ion mobility, ranging from 20 eV at 0.6 V s/cm² to 59 eV at 1.6 V s/cm², using default parameters. Instrument calibration was performed with three ions from the ESI-L Tuning Mix (Agilent, Santa Clara CA USA) at m/z 622, 922, and 1222.

The custom workflow ‘LFQ-MBR’ from FragPipe version 21.1 (fragpipe.nesvilab.org) was used for the analysis of raw data acquired by the mass spectrometer. This workflow includes database searches with MSFragger (version 4.0) and deep-learning prediction rescoring with MSBooster, Percolator, and ProteinProphet (Philosopher version 5.1.0) for validating peptide-spectrum matches and inferring proteins. The raw .d files from Bruker were searched against the *Chlamydomonas* *reinhardtii* v5.6 database (available at phytozome-next.jgi.doe.gov/info/Creinhardtii_v5_6), which was supplemented with CDKL5-GFP, CDKL5^K33R^-GFP, and common contaminant proteins. Decoy reversed sequences were also included in the search database.

Default parameters were used for MSFragger searches, with precursor and fragment mass tolerances set to 20 ppm. Peptide length was set between 7 and 30 amino acids. To study protein phosphorylation, variable modifications included phosphorylation of threonine, serine and tyrosine, oxidation of methionine, acetylation of the protein N-terminus, and dimethylation of arginine while carbamidomethylation of cysteine was set as a fixed modification. Maximum variable phosphorylations and modifications on a peptide were set to 5. For MSBooster, Percolator, and ProteinProphet, default options were employed.

Non-filtered search results were processed for downstream label-free quantitative analysis using Scaffold Q+S (Proteome Software Inc., Portland OR USA, version 5.3.0). Threshold values were set at 95% for peptide identification and 99% for protein identification (with a minimum of two peptides) using the Trans-Proteomic Pipeline from the Institute for Systems Biology [68].

### In-gel protein digestion and TMT-LC-MS/MS analysis for Experiments 8 and 9

Gel slices were subjected to standard trypsin digestion protocol with triethyl ammonium bicarbonate as the buffer. Digested peptides were dried under vacuum and labeled with 6-plex TMT reagents (Thermo Fisher, Waltham MA USA) according to the manufacturer’s protocols. Experiment 8 involving the three biological replicates of wild-type (21gr-1, -2, -3) and mutant (lf5-1, -2, -3) were labeled with 126, 127, 128, and 129, 130, 131, respectively and the samples of Experiment 9 involving lf5 CDKL5^K33R^-GFP-TG, lf5, lf2 CDKL5-GFP, 21gr, lf5 CDKL5^Y166F^-GFP-TG and lf5 CDKL5-GFP-TG were labeled with 126, 127, 128, 129, 130, and 131, respectively. Short MS runs and database searches confirmed satisfactory labeling efficiencies. The reactions were then quenched by hydroxylamine. The combined labeled peptides were fractionated using the high-pH reversed-phase spin column (84868, Thermo Fisher, Waltham MA USA) into 8 fractions. All fractions were dried under vacuum and kept at -80^o^C until MS analysis. 0.5 mg of the combined TMT-labeled peptides were desalted using spin columns (89852, Thermo Fisher, Waltham MA USA) and enriched for phosphopeptides using Fe-NTA phosphopeptide enrichment spin columns (A32992, Thermo Fisher, Waltham MA USA).

The TMT-labeled total peptide and phosphopeptide-enriched samples were analyzed by nanoscale high-performance liquid chromatography on an EASY nLC coupled to the Orbitrap Fusion (for total peptides from Experiment 8) or the Q-Exactive Plus mass spectrometers (for enriched-phosphopeptides from Experiment 8, total peptides from Experiment 9, and enriched-phosphopeptides from Experiment 9). Peptides were separated on a 100-μm capillary column packed with 2.7-μm Halo C18 (Michrom Bioresources Bruker, Billerica MA USA) at a flow rate of 300 nl min^-1^. Peptides were introduced into the mass spectrometer via a Nanospray Flex ion source. For the samples analyzed on the Q-Exactive MS, data were acquired in a data-dependent “Top 10” acquisition mode with lock mass function activated (m/z 371.1012), in which a survey scan was followed by 10 higher-energy collisional dissociation tandem mass spectrometry (MS/MS) scans on the most abundant ions at 35,000 resolution with the scan range starting at m/z 110. MS/MS scans were acquired with an isolation width of 0.7 m/z and a normalized collisional energy of 33%. Dynamic exclusion was enabled. The method for the Fusion MS is configured as follows: “Top speed in 3 seconds” acquisition mode was used where a survey scan was acquired followed by data-dependent collision-induced dissociation MS/MS scans on the most abundant ions in the ion trap with a normalized collision energy of 35% and an isolation width of 0.7 m/z. MS3 was performed using Synchronous Precursor Selection with isolation widths of 3 Da and a nominal collision energy of 65%. The product ions from MS3 were scanned in the Orbitrap with a resolution of 50,000. Dynamic exclusion was enabled. In Experiment 8 samples were injected twice as technical replicates, whereas in Experiment 9, total protein samples were run three times as technical triplicates, and phosphopeptide samples were analyzed in technical quadruplicates.

Mass spectrometry .raw files were analyzed using Proteome Discoverer (PD) 2.5. Fractions from high-pH reversed-phase separation were incorporated as “fractions” into PD. Biological replicates and technical replicates were assigned accordingly. Product ion spectra were searched using SEQUEST in the PD standard processing workflow for TMT experiments against the Creinhardtii_281_v5.6 fasta protein database with the addition of the wild-type and mutant sequences. Search Parameters were as follows: (1) full trypsin enzymatic activity; (2) maximum missed cleavages = 2; (3) minimum peptide length = 6; (4) mass tolerance for precursor ions was set at 10 ppm, and fragment ion mass tolerances were set at 0.02 Da and 0.6 Da for Q-Exactive Plus and Fusion searches, respectively; (5) dynamic modifications on methionines (+15.9949 Da: oxidation), and on serine/threonine/tyrosine (+79.9663 Da: phosphorylation); dynamic modification specific to the peptide terminus included: N-terminus acetylation (+42.011 Da), Met-loss (-131.040 Da), and Met-loss+Acetyl (-89.030 Da). (6) 4 maximum dynamic modifications allowed per peptide; and (7) static carbamidomethylation modification on cysteines (+57.021 Da) and static TMT6plex modification on N-termini and lysines (229.163 Da). Percolator node was included in the workflow to limit the false discovery rate (FDR) to less than 1% in the data set using the concatenated database. The “IMP-ptmRS”, “Peptide Isoform Grouper”, and “Modification Sites” nodes were included in the workflow to calculate site probabilities and to distinguish peptides with multiple modification sites within the same phosphorylated peptide. The abundances of TMT labeled peptides were quantified with the Reporter Ions Quantifier node in the consensus workflow and parameters were set as follows: (1) both unique and razor peptides were used for quantification; (2) Reject Quan Results with Missing Channels: False; (3) Apply Quan Value Corrections: True (values set according to the product spreadsheet (Lot #: WB317368); (4) Co-Isolation Threshold: 75 (Fusion); 50 (Q-Exactive Plus) (5) Average Reporter S/N Threshold = 10; SPS Mass Matches [%] = 65 (Fusion only); (6) Normalization mode: “no normalization”; and (7) Scaling Mode was set “on All Average”. Non-nested design was used for the analysis of biological replicates. Protein ratio calculation was “Protein Abundance Based”. Two-tailed t-test was applied to the 1060 vs 4560 experiment. ANOVA with post-hoc Tukey HSD was used for the WT vs. 5 mutants experiment with the samples in the 3 (total protein samples) or 4 (phosphopeptide enriched samples) individual runs treated as replicates. Adjusted p-values were calculated by Benjamini-Hochberg procedure. All the protein identification and quantification information (<1% FDR; with protein grouping enabled) was exported from the Proteome Discoverer result files to Excel spreadsheets.

### Downstream Data analysis for CDKL5 phosphorylation analyses from Experiments 3 to 7

To further analyze the MS data using the label-free quantitative analysis method, data was exported from Scaffold files into Excel files where all the calculations were done.

To export data from Scaffold files, peptide probability cutoff was set at 100%. The sum of the intensities of the precursors for each phosphoisoform including the non-phosphorylated form of each peptide were calculated for each sample and was defined as intensities for phosphoisoform. The intensities from the strains lf2 CDKL5-GFP and lf5 CDKL5^K33R^-GFP-TG were then normalized against those from the strain lf5 CDKL5-GFP-TG using total intensities from all the precursors for CDKL5 proteins from these strains. The protein sequence used to number putative phosphorylation sites is shown in S1 Table. Each dataset was manually examined to identify any abnormalities. To focus on the details of the phosphorylation states of the peptide (aa 155-170) that contains the activation loop, the phosphoisoforms of this peptide and their corresponding normalized intensities were copied from the above calculated datasets (S2 table, sheet “All CDKL5 peptide isoforms”) and are plotted in Fig 5A for direct comparison. To illustrate that the peptide 155-170 is readily detected in *lf2* samples but not in wild-type or CDKL5^K33R^ samples, the sum of the normalized intensities of all phosphoisoforms, whether phosphorylated or not, was calculated for every CDKL5 peptide. The average ratios from Experiment 3 and 4 for each peptide of lf2 CDKL5-GFP to lf5 CDKL5-GFP-TG or of lf2 CDKL5-GFP to lf5 CDKL5^K33R^-GFP-TG were calculated (S2 table, sheet “All CDKL5 peptides”). The average ratio was plotted according to the peptide’s start position on CDKL5in Fig 5B. Similar analyses were done for Experiments 5, 6, and 7. Data from Experiments 6 and 7 (S2 table, sheet “CDKL5 (WT & K33R) CIP peptides) are plotted in Fig 5C and D.

### Downstream data analysis for proteomic and phosphoproteomic analyses from Experiments 8 and 9

For total protein analysis, any protein with an abundance ratio of *lf5* sample to wild-type control sample smaller than 1 and adjusted p-value for the abundance ratio smaller than or equal to 0.05 was considered reduced in the *lf5* sample. Any protein with an abundance ratio of *lf5* sample to wild-type control sample larger than 1 and adjusted p-value for the abundance ratio smaller than or equal to 0.05 was considered increased in the *lf5* sample. Only the proteins considered increased or reduced in both biological sets of samples were reported as increased or reduced in the final result.

For phosphoproteomic analysis, the adjusted abundance ratio (phosphopeptide abundance ratios / their corresponding protein abundance ratios) of the *lf5* mutant sample to the wild-type control sample for every phosphorylated peptide was first calculated. For phosphopeptides whose corresponding protein levels (Experiment 8) were not changed in the *lf5* mutant sample (i.e. adjusted p-value for the protein was larger than 0.05), the phosphopeptide abundance ratios were not adjusted. For peptides whose corresponding protein levels changed in the *lf5* mutant sample (i.e. adjusted p-value for the protein was smaller than 0.05), the adjusted abundance ratio for the phosphopeptide equals the abundance ratio for the phosphopeptide divided by the abundance ratio for the corresponding protein. Any phosphorylated peptide with an adjusted abundance ratio smaller than 1 and adjusted p-value for the abundance ratio smaller than or equal to 0.05 was considered to have reduced phosphorylation in the lf5 sample. Only the sites that were found to have reduced phosphorylation in both biological sets of samples were identified as having reduced phosphorylation in the result (S4 Table, sheet “All Positives”). Among these sites, sites that had an adjusted abundance ratio smaller than 0.549 in both biological sets of samples (S4 Table, sheet “Positives 2 Fold”) were used to deduce the CDKL5 phosphorylation consensus motif. Five amino acids in front of and after the identified sites were acquired from the proteins’ sequences (S4 Table, sheet “Motif Analysis”). At each position, the percentage of occurrence of each amino acid among sequences under consideration was calculated (“percentage among 43 sites”). Meanwhile, the full-length sequence of all the proteins that contain reduced phosphorylation sites were acquired. The percentage of occurrence of this same amino acid within these sequences was calculated (“percentage among all positive proteins”). Fold change in abundance was calculated by dividing the percentage among 43 sites with the percentage among all positive protein sequences. The data were plotted using Excel to visualize a pattern (Fig 11B).

### Downstream data analysis for identification of CDKL5-interacting proteins from Experiments 10 and 11

First, all the proteins identified in the datasets were filtered according to the peptide threshold, protein threshold, and the minimum number of peptides identified for each protein. For Experiment 10, peptide threshold was set at 99.5% minimum with peptide False Discovery Rate (FDR) at 0.1%; protein threshold was set at 99.9% minimum and at least 2 peptides identified with protein FDR at 1.2%. For Experiment 11, peptide threshold was set at 99.9% minimum with peptide FDR at 0.0; protein threshold was set at 99.9% minimum and at least 2 peptides identified with protein FDR at 1.9%. All the proteins that met these criteria were exported from Scaffold files to Excel files with their total precursor intensities for further analyses. For Experiment 10, data from the three technical replicates with the same loading amount (1 µl) were used for analysis. Next, each dataset went through data cleaning to filter out proteins without quantitative values for total precursor intensity in any of the technical replicates, followed by transformation to log_2_, imputation to fill the blank cells where no quantitative value was available, and calculation of ratio and p value [69]. Then, the two datasets were combined (S5 Table sheet “All Proteins”) and the following criteria were applied to screen out positive proteins in each dataset: 1. ratio of total precursor intensity from experimental sample to that from lf5 control sample larger than 10; 2. p-value smaller than 0.01. Proteins that were positive in both datasets were deemed candidates for CDkL5-interacting proteins (S5 Table sheet “Positive Proteins”).
