# Supplementary material for: Activation of the ciliary kinase CDKL5 is mediated by the cyclin-dependent kinase CDK20/LF2 to control flagellar length
Source: PLoS Biol. 2025 Dec 12;23(12):e3003560. doi: 10.1371/journal.pbio.3003560 (PMC12711092; doi:10.1371/journal.pbio.3003560)
Supplement: Attachment — Submitted filename: Rebuttal_09_26_2025.docx [file pbio.3003560.s031.docx]

REVIEWS:

Reviewer #1: CDKL5 is part of a family of evolutionarily ancient kinases distantly related to cyclin-dependent kinases. CDKL5 in particular has attracted considerable attention due to its association with Cdkl5 Deficiency Disorder (CDD), a neurodevelopmental disorder characterized by epileptic seizures with an incidence of ~1:40,000, rare but considerably more common than eg Bardet-Biedl Syndrome (BBS, 1:140,000), a classical ciliopathy that is moreover genetically heterogeneous, with mutations in >20 loci. CDKL5 was originally linked to cilia and ciliary length control in a study in Chlamydomonas from 2013 (Tam et al., Mol Biol Cell). Further work by Mustafa Sahin and Cecilia Lo's labs in 2022 (Di Nardo et al., Neurosci Res; Faubel et al., Acta Neuropathol) showed that motile cilia in both CDD patients and knockout mice were elongated and poorly motile, with impaired cilia-driven flows in the mouse brain inducing seizure-like activity. Despite these clear data linking cellular function and clinical presentation and further indications of a role in ciliary length control for this family of kinases from studies on the paralog CDKL1 in C. elegans (Park et al., Curr Biol 2021), only 7 of the 776 largely clinical studies on CDKL5 to be found on PubMed make any mention of cilia in their title or abstract, highlighting a disconnect between applied research and basic cell biology largely conducted in model organisms. Further work on this kinase with a view to better understand its ciliary function is therefore clearly welcome.

The present study by the labs of Karl Lechtreck, George Witman and Gregory Pazour represents a follow-up on the work by Tam et al., further characterizing CDKL5/LF5 and its link to another kinase also involved in ciliary length control, CDK20/LF2 (Tam et al., J Cell Biol 2007), both in Chlamydomonas and to a lesser extent also in mice. The authors report that CDKL5 localization and function in ciliary length control in Chlamydomonas requires its kinase activity and that LF2 phosphorylates CDKL5's activation loop to regulate its kinase activity. Vertebrate CDK20 reportedly similarly regulates CDKL5 localization to the ciliary base in mice. Finally, the authors perform differential ciliary proteomics from wild-type and CDKL5 mutant Chlamydomonas and CDKL5 immunoprecipitations, identifying IFT proteins as increased in abundance in loss of function mutants and FAP93 as a potential regulator of CDKL5 localization to the ciliary base. Most intriguingly, the authors report CDKL5 phosphorylation of a residue within the IFT74 tubulin-binding domain, providing a potential molecular mechanism by which the kinase could control ciliary length.

Overall, then, there is plenty of data within the manuscript's sprawling 12 figures and 5 supplemental figures and experiments on the whole are conducted to a reasonable standard. My main criticism is that the manuscript feels like a grab bag of disconnected experiments, written in a way that is not immediately accessible to the reader. There is no obvious storyline. If published in its present format this would not serve its stated cause to raise the profile of CDD as a potential ciliopathy.

We appreciate the reviewer for their thorough analysis of our manuscript.

Main points

1. Clarity. Throughout the manuscript the authors use unnecessarily complicated language, making it extremely difficult to follow their arguments. Experiments are also presented in a highly idiosyncratic manner. Just to highlight a few examples:

We have taken this reviewer’s comments seriously and made extensive edits to the text to clarify the experiments. We had originally used extensive wording to describe the cell lines used in each experiment but now realize that this made the text difficult to comprehend. We have streamlined the examples pointed out by the reviewer and have made extensive edits throughout the document to address this issue.

p5/6

The results would appear to start with examining the phylogenetic distribution of CDKL kinases (p5) or at the latest when examining CDKL5 localization in motile and non-motile cilia (p6), yet this paragraph and the entirety of Fig 1 is first discussed in the Introduction (although the authors return to this theme on p8). Why?

The description of the phylogenetic distribution of CDKL kinases and Fig 1 have now been moved to a new subsection (“Phylogeny of CDKL5 and related kinases”) at the beginning of the Results section. This is indeed a more appropriate location for this material.

p8/9

"Mammalian CDKL5 localizes to cilia and defects in it cause abnormally long cilia" (p8).."These data indicate that CDKL5's role in cilia length control is conserved in mammalian primary cilia." (p9) The only novel aspect here is that the kinase dead mutant does not rescue, yet this aspect is not highlighted.

This has been reworked to place the emphasis on the kinase activity. It now reads

“Overexpression of the wild-type gene reduced the cilia length to slightly less than normal. Expression of kinase-dead Cdkl5^K42A^ did not restore cilia length indicating that kinase activity is fundamental to Cdkl5’s function in regulating cilia length. Expression of Cdkl5^T169A,Y171F^ with mutated activation-loop phosphorylation sites was able to partially rescue although not as well as the wild-type construct. (Fig 3B, 3C).”

p12

For the most part, the phosphorylation patterns of CDKL5-GFP expressed in a lf2 background (i.e. lf2 CDKL5-GFP cells) and CDKL5K33R expressed in a lf5 background (i.e. lf5 CDKL5K33R-GFP-TG cells) were similar to that for CDKL5-GFP expressed in a lf5 background (i.e. lf5 CDKL5-GFP-TG cells) Can this not be expressed in simpler terms?

We apologize for the unnecessary complexity. This passage now reads:

“For the most part, the phosphorylation pattern of CDKL5 was similar in all three strains (S2 Table).”

p14

"Ability of CDKL5 to autophosphorylate in vitro is dependent upon in vivo expression in the presence of LF2 but not LF4"

What is this title supposed to mean?

The title has been changed to “LF2 is required for CDKL5’s kinase activity”

p17

"To test the involvement of Cdk20 in regulating Cdkl5 localization, we removed Cdk20 from Cdkl5 mutant fibroblasts and rescued with the Cdkl5-6xMyc, Cdkl5K42R-6xMyc, and Cdkl52A-6xMyc constructs."

It is a little strange to refer to the expression of an unrelated GFP transgene as a 'rescue'.

Rewrote to explain that Cdkl5 is being rescued in a double mutant line.

“To test the involvement of Cdk20 in regulating Cdkl5 localization, we removed Cdk20 from Cdkl5-mutant fibroblasts (S6 Fig) and rescued the Cdkl5 mutation with the Cdkl5-6xMyc, Cdkl5^K42A^-6xMyc, and Cdkl5^T169A,Y171F^-6xMyc constructs (Fig 8).”

p17

"To determine if mammalian Cdkl5's ciliary localization is regulated like Chlamydomonas CDKL5, we mutated the active site (Cdkl5K42R) and mutated the two phosphosites in the activation loop (Cdkl52A)."

The 2A mutant already appeared on p9, where it was not properly introduced ("Overexpression of the wild-type gene reduced the cilia length to slightly less than normal whereas expression of kinase-dead Cdkl5 did not alter cilia length but expression of Cdkl5 with mutated activation-loop phosphorylation sites was able to rescue. (Fig 3C)").

Cdkl5^2A^ has been renamed Cdkl5^T169A,Y171F^ throughout the document and introduced at first use on p10. Similarly, CDKL5^3A^-GFP has been changed to CDKL5^S162A,T164A,Y166A^

Text has been adjusted in the two places to more appropriately describe the experiments.

p19

"In support of this, the pattern for CDKL53A-GFP was much closer to that of CDKL5K33R-GFP than to CDKL5Y166F-GFP: compared to full-length lf5 CDKL5-GFP-TG flagella, CDKL53A-GFP transport by IFT was strongly upregulated, there was much less accumulation of the protein at the flagellar base, and there was some accumulation at the flagellar tip (Fig 9E, F)."

It is almost impossible to parse this sentence.

This section has been rewritten.

“Supporting this interpretation, CDKL5^S162A,T164A,Y166A^ behaved more like CDKL5^K33R^ than CDKL5^Y166F^. Importantly, in steady-state flagella, CDKL5^S162A,T164A,Y166A^ transport was strongly upregulated compared with wild type and failed to undergo the tip-to-base redistribution characteristic of wild-type protein (Fig. 9E, F).”

p21

"The site was phosphorylated in CDKL5-GFP from whole-cell lysates of lf5 CDKL5-GFP-TG cells but not from lf2 CDKL5-GFP cells, and not in CDKL5K33R from lf5 CDKL5K33R -GFP-TG cells, indicating that phosphorylation at this site occurs by autophosphorylation following activation of CDKL5 by LF2 (S2 Table and see section "Phosphorylation at the CrCDKL5 activation loop is controlled by LF2 and does not depend on autophosphorylation")."

Again, much more complicated than necessary.

We have rewritten the text to now say

“The site was phosphorylated on CDKL5-GFP isolated from control cells but not on protein isolated from a *lf2* mutant, and not on kinase-dead CDKL5, indicating that phosphorylation at this site occurs by autophosphorylation after CDKL5 is activated LF2.”

Fig S1B (now S2)

Why label a blot 11479.6T and 26548 1T-6T rather than Control and KO lines 1-6?

Firstly, they are not all KO lines so that would be incorrect. 26548 1T-6T are lines derived from the embryos of one litter that is segregating the CDKL5 deletion. Secondly, we get a lot of requests for cell lines and having the ability for investigators to ask for the exact line is important.

To clarify we have added the words “Control” and “Mutant” to indicate which cell lines were used in subsequent experiments.

Fig S1C (now S2 and S3)

Why show panel C, functionality of GFP CRISPR Chlamydomonas lines, in the same figure as panel B, characterizing CDKL5 mutant MEFs?

These have been split into separate supplemental figures.

Fig 3B/C

There is no need to label an image panel with a plasmid number (eg MS234) which is nowhere referred to in the main text. Also -/y might be technically correct, but could simply be described as - with the hemizygous nature clarified in the legend. Finally, labeling one transgene K42A, the other 2A is confusing when the latter refers to T169A,Y171A.

Plasmid names have been removed and Cdkl5^2A^ has been renamed Cdkl5^T169A,Y171F^

Fig S2B/C

Why label knockout MEF cell lines and rescue condition in a way that is not accessible to the reader?

The figure has been remade to clarify the genotypes of the cells.

Fig 4A

This schematic fails to show phosphosites or any other feature of the protein (eg kinase domain) in a way that would be accessible to the reader.

We have now replaced Fig 4A with an improved graphic that shows the kinase domain, the lysine at the catalytic site, and specific residues that are phosphorylated throughout the protein.

Fig 5 (and S3)

This appears a sub-optimal way to present mass spec data. If the intent is to allow the reader to compare the levels of specific (phospho-)peptides in different experimental conditions, this is not easily done from either the tables or dot plots.

We thank the reviewer for pointing out the difficulty in comparing the levels of specific phosphopeptides under different experimental conditions in original Figures 5 and S3.

We have replaced the old figures with new graphics. The tables in old Fig5Aa and Fig5Ba/c have been replaced with bubble charts where the amount of peptide correlates with the bubble size. The multiple complicated dot plots in Fig5Ab and 5Bb have been replaced with single dot plots showing the ratio of the peptide abundances in the conditions under comparison. This greatly simplifies the presentation and more strongly makes our point.

S3 became unnecessary with the new approach and has been removed.

Fig 10D

Why invert the bar graphs?

We chose an inverted bar graph for Fig 10D to show *decreases* in specific proteins in *lf5* flagella compared to WT flagella. Visually, this contrasts with the normal bar graph in Fig 10B showing *increases* in other proteins. The use of inverted bar graphs to emphasize decreases in values is not uncommon and has been used previously by us and others (e.g., Picariello et al., 2019; Engel et al., 2012).

Fig 11B

Potential substrate recognition motifs are not usually shown in this manner. Sequence logos would be much more interpretable.

To address this reviewer’s concern, we have added a traditional sequence logo to the figure.

2. Mechanistic insight/Novelty. In their abstract and concluding paragraph, the authors highlight their findings of CDKL5 localization to cilia and defects in ciliary length and motility in CDKL5 mutants and conclude that their results provide "..strong evidence that CDKL5 dysfunction contributes to ciliopathy-related phenotypes, supporting the hypothesis that CDD is a ciliopathy." (p33) As mentioned above, CDKL5 localization and ciliary length and motility phenotype has been previously reported, both in Chlamydomonas and in mice. Furthermore, there is quite convincing data supporting impaired ciliary motility in the brain as the underlying cause of the epileptic seizures in CDD patients, which goes far beyond what is shown in this manuscript. What is at least to some extent novel in their manuscript is the following:

A review of the literature shows that most CDKL5 investigators do not consider CDKL5 to be a ciliary protein.

1) CDKL5 kinase activity is shown to be required for its function - although this is perhaps unsurprising, given that this has been previously shown to be the case for CDKL-1 in C. elegans (Canning et al., Cell Rep 2018).

CDKL-1 is a different protein. While it is expected that kinase activity is important for a protein kinase, our work also shows that CDKL5 kinase activity controls both its transport by IFT and its localization in cilia, which are novel observations.

2) LF2 phosphorylates CDKL5 within the activation loop and thereby regulates its kinase activity - this is perhaps the most substantive finding in this manuscript, although again perhaps unsurprising, given that LF2 was shown by Tam et al., 2013 to affect CDKL5 localization in Chlamydomonas and CDK20 knockouts display elongated cilia with an accumulation of IFT proteins at their tips in RPE1 cells (Noguchi et al., PLOS One 2021 - incidentally a paper not cited by the authors).

This IS surprising and novel! There are lots of ways that the loss of LF2 could affect the localization of CDKL5, such as the generation of docking sites for CDKL5. Our finding that the activation-loop phosphorylation is dependent on LF2 is not predicted by any studies in the literature. The literature predicts that autophosphorylation is key, which we show is not the case.

The Ift88 accumulation phenotype was originally reported in Snoufer et al 2017 (PMID: 28817564), which we had cited. We have added a citation to the Noguchi paper.

3) Active CDKL5 in Chlamydomonas localizes to the ciliary base of mature cilia, whereas inactive kinase is unmoored and accumulates along cilia and at their tips, as it normally does in regenerating cilia - this had previously been shown for the wild-type protein by Tam et al. in their 2013 study; that the inactive kinase remains delocalized is interesting; however, the functional significance of this observation is unclear since it is perhaps not conserved in vertebrates (Fig 8) and delocalization in FAP93 mutants does not impact flagellar length (Fig S5).

Indeed, Tam et al. showed that the distribution of CDKL5 changes as cilia mature and we have cited their work repeatedly in the present manuscript. Our new finding is that kinase activity is required for this redistribution and also regulates the association of CDKL5 to IFT. Furthermore, the kinase activity cannot be supplied in trans.

4) Differential proteomics and IP interactomics identifies potential CDK5L targets/regulators - although these are either not further characterized or as in the case of FAP93's tethering function apparently dispensable for CDKL5 function. Regarding the ciliary accumulation of IFT proteins in CDKL5 mutants, something similar was shown for CDK20 mutants by Noguchi et al., where this was interpreted as impaired retrograde IFT, so again not entirely unprecedented.

While CDKL5 is undoubtedly a clinically relevant protein, the added mechanistic insight here in my view is limited. What to me would strengthen the manuscript considerably is a better understanding of the relationship between CDKL1 and CDKL5, which seem to perform the same function but are not redundant. Are the phenotypes additive? Similarly, is CDK20 merely an upstream regulator of CDKL5 (and 1?)? If so, there should again not be an additive phenotype in double mutants. These were actually generated by the authors (Fig 8), but their ciliary lengths were not assessed. Is retrograde IFT impaired? The authors have the tools to address these questions, which would actually advance our understanding of the function of this kinase family.

While we agree that many questions remain we feel that this summary overlooks the key mechanistic insights provided by our work. Novel findings include:

1) the discovery that CrCDKL5 is activated by the protein kinase LF2, raising questions about the current dogma that mammalian CDKL5 is activated by autophosphorylation of an activation-loop tyrosine

2) the discovery that the kinase activity of both CrCDKL5 and mammalian Cdkl5 is necessary for the protein’s normal localization (a result, incidentally, in contrast with the findings for CeCdkl-1)

3) the discovery that intraflagellar transport protein IFT74 is a target of CDKL5, and that phosphorylation of IFT74 at the target site would be expected to down regulate IFT transport of tubulin into the flagellum, explaining why loss of CDKL5 causes an *increase* in flagellar length

4) identification of the protein responsible for tethering CrCDKL5 to the proximal axoneme, leading to a hypothesis for why *Chlamydomonas* has evolved this unusual mechanism for retaining most CDKL5 in the flagellum

5) the observation that the activity of CrCDKL5 regulates its association with IFT, showing that a cargo regulates its own transport

These important findings, new hypotheses, and new questions yet unanswered, will provide a solid foundation for future work on both mammalian and *Chlamydomonas* CDKL5 and related kinases.

Other points

3. Characterizing the lf5 mutant

The authors describe the lf5 mutant as a null mutant (eg p7). This mutant was originally generated by Tam et al., 2013 (there referred to as DKD6 or lf5-2). In that puplication the authors failed to detect any transcript, but also did not map the genomic alteration. If there is residual expression of a truncated version of the protein or the mutation takes out more than just CDKL5, this would throw the failure to fully rescue the mutant using a GFP transgene (eg Fig. 2C, D) into a different light. Have the authors considered either sequencing the lf5-2 mutant or generating a clean gene deletion?

We thank the referee for raising this important point. Mapping of the lf5-2 allele has been completed and the information has been added as S1 Data. We found that the 5’end of CDKL5 is deleted, which removes the kinase domain. Tam et al. did not detect a transcript by northern blot making it unlikely that a truncated protein is being produced at any significant level. The deletion does take out two upstream genes. Neither of these have been identified in any study of flagella and they are not highly conserved, making it unlikely that they are crucial to ciliary function.

Accordingly, we have added a caveat to the results noting that two additional unknown genes are removed in this allele.

“Our mapping of the deletion that created the lf5-2 allele revealed that two upstream genes were also deleted (S1 Data). Both are uncharacterized. One is predicted to be a sucrose 6-glucosyltransferase and the other a ribosome-binding GTPase. Neither have been connected to flagella in any proteomic or other study, making their importance to the regulation of beat frequency unknown.”

4. Characterizing the vertebrate MEF knockouts While the authors do show blots to confirm full-length CDKL5 is no longer expressed in their MEF cell line knockouts (Fig S2) I could not find any such blot or IF images for CDK20. There is also no information on which exons were targeted for either CDKL5 or CDK20. They do report the sequences of the guides in Table S8, but one would expect at least a schematic showing where these are in the gene, also relative to the epitopes of the commercial antibodies the authors used to detect the two proteins.

This has been added as S6 Figure.

5. CDKL5 localization in mice

p6 "Similar to what was observed in Chlamydomonas, mouse Cdkl5 localizes to both non-motile primary cilia and to motile cilia, although we did not observe the prominent enrichment at the proximal end of mammalian cilia that is observed in Chlamydomonas (Fig 1C, D)." This finding, also highlighted in the abstract along with the depletion phenotype, was previously shown by Faubel et al. Moreover, if the authors want to make something of the apparent discrepancy with protein localization in Chlamydomonas or humans (Canning, Cell Rep 2022), they would need to demonstrate antibody specificity by staining their knockout MEFs.

We are not sure that we understand what the reviewer is asking. Faubel’s observations were cited in several instances. Canning et al. do not show label at the proximal end of the cilium in a position analogous to the site in *Chlamydomonas*. We have now added staining of KO MEFs with the CDKL5 antibody to S2 Fig.

5. CDKL5 and the CDKL family of kinases

On p5, the authors state "The Chlamydomonas genome encodes two proteins, CrCDKL5 and FAP262, that are similar to the CDKLs." While this may be true, their Fig 1A fails to make this point clearly. If this phylogenetic tree is to be taken at face value, Chlamydomonas encodes four other CDKL-related proteins, FLS1, FLS2, CrCRE09.g395658 and CrCe17.g709500, which are more similar to human CDKL1-4. This is inconsistent with the proposed evolutionary history of the CDKL protein family recently published by Martín-Carrascosa et al., Front Cell Dev Biol 2025 according to which chlorophytes possess only the ancestral CDKL5 protein (their Fig 2), a paper that incidentally would seem well worth citing. If these additional proteins are unrelated, a better tree ought to be shown. If not, and in particular given the similar function in ciliary length control reported for CDKL1 in C. elegans, their potential function ought to be examined.

It is unclear why the 5 proteins that we included in the tree were not examined in the Martín-Carrascosa et al. paper. They state “We performed systematic BLASTP searches using the sequences of the five human CDKLs as queries. When we identified the homologous proteins in the species of interest, we performed the reverse procedure; each candidate was used as a query against human proteins and checked that the best match was a CDKL protein; and those that returned a different protein, usually a CDK, were discarded.”

Using these criteria, we find that blasting the *Chlamydomonas* genome with human CDKL5 retrieves

CDKL5 (Cre12.g538300.t1.1)	BLAST E value 1.50e-119	Reverse BLAST HsCDKL5 1e-127

FAP262 (Cre03.g164250.t1.1)	BLAST E value 4.25e-74	Reverse BLAST HsCDKL5 3e-85

FLS2 (Cre03.g169500.t1.2)	BLAST E value 1.31e-72	Reverse BLAST HsCDKL1 7e-97

FLS1 (Cre12.g551250.t1.2)	BLAST E value 2.95e-64	Reverse BLAST HsCDKL1 3e-69

Cre09.g414650.t1.1	BLAST E value 6.22e-60	Reverse BLAST HsCDKL1 5e-75

Cre17.g709500.t1.1	BLAST E value 2.22e-49	Reverse BLAST HsCDKL1 4e-61

which clearly fit their criteria.

We contacted the senior author of the Martín-Carrascosa et al. paper to ask for further details of why they did not include these proteins but did not get an answer to that question. They suggested that we include these sequences in the larger set of CDKLs that they analyzed. We did this and obtained a tree too large to include in the manuscript, but all of the major branches remained in the same place with FAP262 and CrCDKL5 grouping with mammalian CDKL5 and the FLS1, FLS2, CrCre09.g395658 and Cre17.g709500 family forming a sister group to mammalian CDKL1/2/3/4, CeCDKL-1, and DmCDKL. Furthermore, the two papers on FLS1 and FLS2 called these CDKL proteins and produced trees equivalent to ours. For example, see Figure 1E pasted below from “FLS2 is a CDK-like kinase that directly binds IFT70 and is required for proper ciliary disassembly in Chlamydomonas” Zhao et al. Pan. PMID: 32134924 PMCID: PMC7077844 DOI: 10.1371/journal.pgen.1008561.

Drosophila CDKL has now been added to the tree and the Martín-Carrascosa et al. paper has been cited.

This section has been rewritten to more carefully describe the relationship between the CDKLs from the various species.

Minor comments/text changes

p4

"Cilia and flagella (terms can be used interchangeably)"

I might be a little overly pedantic here, but while a flagellum is a type of cilium, not every cilium is a flagellum.

Most would state the opposite as bacterial flagella are not cilia. In eukaryotes, knowledgeable investigators see the distinction as historical and not based on biochemical or structural parameters. So as not to distract the reader from our main points, we have changed the phrase to “Henceforth we will use the terms “flagella” when referring to *Chlamydomonas* and “cilia” when referring to mammals or making a generalization” and moved it to lower in the paragraph. We trust no one will find fault with this wording.

p8

"Similarly, in contrast to the partial rescue of swimming speed observed with wild-type CDKL5-GFP, neither of the mutated proteins rescued swimming speed (Fig 2C), indicating that CDKL5's kinase activity is necessary for normal motility. Consistent with the results for wild-type CDKL5-GFP, expression of the mutated proteins had little or no effect on beat frequency (Fig 2D)."

Contrary to what the authors imply, Fig 2D shows the two mutant rescues to have beat frequencies similar to the wild-type rescue, all of which are slightly improved over the lf5 mutant alone but none near that of controls.

We agree and have rewritten the sentences as follows:

“Unlike wild-type CDKL5, the mutant proteins did not restore normal flagellar length or swimming speed (Fig. 2A, 2C), showing that CDKL5’s kinase activity is essential for regulating flagellar length and motility. As expected, given the failure of wild-type CDKL5 to fully rescue beat frequency, the mutant proteins resulted in little (CDKL5^Y166F^) or no (CDKL5^K33R^) rescue (Fig 2D).”

p9

"Overexpression of the wild-type gene reduced the cilia length to slightly less than normal whereas expression of kinase-dead Cdkl5 did not alter cilia length but expression of Cdkl5 with mutated activation-loop phosphorylation sites was able to rescue. (Fig 3C)."

The statistical tests in Fig 3C should assess rescue ie compare to the mutant, not to wild-type. Further, the surprising finding that the activation loop mutant is able to rescue is not further remarked upon, even though just one paragraph later the authors comment on how important this phosphorylation is in humans (based on the literature) and Chlamydomonas (their findings, Fig 2)

The requested statistical comparison has been added to Fig 3C. Using the comparison to mutant as suggested by the reviewer shows that all three constructs are able to rescue to some degree. When the comparison is made to wild-type rescue, the kinase-dead and activation loop mutants fail to function as well as wild type. Changes have been made to the text to include these observations.

p17

"Normal localization of mammalian CDKL5 is independent of its kinase activity but requires Cdk20"

This is somewhat counterintuitive. If CDK20 function is to activate CDKL5 kinase activity and it does so by phosphorylating the kinase on its activation loop, why does loss of CDK20 affect CDKL5 localization, but loss of CDKL5 kinase activity does not?

It is possible that Cdk20 phosphorylates CDKL5 residues in addition to those at the activation loop, and that these other phosphoresidues are involved in maintaining normal localization of CDKL5. This has been acknowledged by adding the following text:

“Interestingly, the loss of Cdk20 had a greater effect than the loss of Cdkl5 kinase activity, suggesting that Cdk20 has an additional role in localizing Cdkl5 beyond activating its kinase activity.”

p18

"To determine if IFT of CDKL5 is dependent on its kinase activity, we similarly observed full-length flagella of lf5 cells expressing CDKL5K33R-GFP, CDKL5Y166F-GFP , or CDKL53A-GFP (Fig 9B, C, E)."

Somewhat misleading. 'Full-length' implies cilia to be morphologically normal, which in the mutant rescues they are not. "Mature" maybe?

We understand the reviewer’s point. Accordingly, throughout the manuscript text and figures, we have changed “full-length” to “steady-state,” which is frequently used to describe a flagellum that has reached the stage where assembly and disassembly are balanced and flagellar growth has leveled off. We hesitate to use the term “mature” to describe a flagellum in which growth has leveled off, yet IFT of CDKL5 remains elevated (as in *lf5* cells expressing CDKL5K33R-GFP, Fig. 9B).

p19

"The movement of CDKL5Y166F-GFP by IFT, together with CDKL5Y166F-GFP's concentration (albeit diffuse) at the flagellar base, suggests that CDKL5Y166F-GFP retains some kinase activity."

This seem a little bit of a stretch, to go from localization pattern to predictions of kinase activity.

We revised this paragraph to more clearly describe our logic:

“Steady-state IFT of CDKL5^Y166F^ more closely resembled wild-type CDKL5 than CDKL5^K33R^, although diffusion appeared more prominent (Fig. 9C, 9F). Unlike CDKL5^K33R^, a substantial pool of CDKL5^Y166F^ accumulated at the base of the flagellar shaft, though in a more diffuse pattern than wild type, and little or none localized to the tip (Fig. 9C). This combination of IFT-based transport and diffuse basal enrichment suggests that CDKL5^Y166F^ retains partial kinase activity. Supporting this interpretation, CDKL5^S162A,T164A,Y166A^ behaved more like CDKL5^K33R^ than CDKL5^Y166F^. Importantly, in steady-state flagella, CDKL5^S162A,T164A,Y166A^ transport was strongly upregulated compared with wild type and failed to undergo the tip-to-base redistribution characteristic of wild-type protein (Fig. 9E, F).

p29

"Forty-two proteins were reduced in abundance in the absence of CDKL5. Some of these proteins have functions related to flagellar motility, providing a possible explanation for the reduced flagellar beat frequency observed in the lf5 mutant. Also of note, the length-regulatory kinase LF4 was reduced, suggesting that LF4's role in IFT is downstream of CDKL5."

While it is tempting to speculate that LF4 may be downstream of CDKL5, the mass spec data of the authors is not particularly conclusive evidence, especially considering Wang et al, FASEB 2019 showed LF4 localization to be normal in lf5 mutants.

We revised the last sentence to place our results in context of previous work:

“Previous studies have shown that LF4 is phosphorylated by LF2, but apparently not by CDKL5, and that LF4 is highly enriched in the flagella [61]. Taken together, these results suggest a complex interplay between CDKL5 and LF4 to regulate flagellar length.”

Reviewer #2: This a well-written, compelling, tour de force on the regulation, localization, and function of a protein kinase that regulates ciliary/flagellar length in Chlamydomonas and vertebrates. Each of the many conclusions in the abstract about CDKL5 are solidly supported by one or more independent experimental strategies and approaches. After decades of research, the molecular mechanisms that regulate cilia/flagella length remain poorly understood. The findings in the manuscript make clear the challenges of investigating length control mechanisms: Length control depends on an exquisite interplay of multiple complex systems. These authors elegantly show that the length-regulating flagellar protein kinase, CDLK5, is phosphorylated at multiple sites through autophosphorylation and through the activity of at least two other protein kinases, and that alterations in CDLK5 phosphorylation influence its location, protein kinase activity, and function. Their comparisons

between the vertebrate and Chlamydomonas proteins support common mechanisms of regulation, and the finding that a tubulin-transporting IFT protein is a substrate for CDLK5 provides a reasonable explanation for a central function of CDLK5 in length control.

This is a beautiful manuscript! I have 3 minor comments:

Thank You!

1) In the Figure 2 kinase activity experiments, it would be helpful to provide the results (shown later) that the mutant forms of the protein are expressed and present in flagella.

This has been added as S3A Fig.

2) Figure 4 A should be made much larger. It shows important results that are well presented in the text but challenging to understand by viewing the figure.

Fig 4A has now been replaced with a snake diagram that shows the kinase domain, the lysine at the catalytic site, and specific residues that are phosphorylated throughout the protein.

3) The Discussion could be shortened. Although well written and easily understood, it sometimes devotes too much text to re-describing results.

The Discussion has been rewritten to be more concise and to eliminate unnecessary repetition of findings presented in the Results section.

Reviewer #3 (Tomoharu Kanie, identifies himself): CDKL5 is a protein kinase that belongs to cyclin dependent kinase family like family. CDKL5 mutations are found in Cdkl5 Deficiency Disorder (CDD), of which phenotypes show overlaps with the ones caused by cilia defects (i.e., ciliopathies). The molecular mechanisms underlying the defects caused by CDKL5 are not well understood. In this manuscript, the authors carried out detailed analyses of Chlamydomonas CDKL5, and its mammalian orthologue, and provide deep insights into the functions and regulations of CDKL5, which include the ciliary waveform regulations, the length control of mammalian primary cilia, comprehensive determination of phosphorylation sites within CDKL5, IFT modulations, and identification of potential substrates. The main texts are logically written, the data is of high quality, interpretation of data is mostly reasonable, statistical analyses were adequately performed, and legends and methods sections provide sufficient details. This paper will be of interest to biologists who work on cilia and flagella as well as geneticists who studies cilia-related disorders.

I think the manuscript is already in a good shape and should be published with only minor modifications. I have only a couple of minor comments for the data interpretation, and this should be solved by modification of the text rather than additional experiments.

Thank you!

Minor comments for the data

I was convinced by almost of all data except the ones that showed phosphorylation of CDKL5 activation loop is mediated by LF2. In Figure 5, the authors showed that peptides with S162/T164/Y166/S168 phosphorylation were almost undetectable in either wild-type or kinase-dead (K33R) CDKL5, while mono-phosphorylation at each of the above residues were found in CDKL5 purified from the alga lacking LF2. The authors hypothesized that CDKL5 may be highly phosphorylated at S162T164Y166 in wild-type cells and that the tri-phosphorylated form may not be detected by mass spec. To test the hypotheses, the authors performed mass spec analyses of CDKL5 before and after the phosphatase treatment, and found that the intensity of phosphopeptides as well as non-phosphopeptides were dramatically increased after CIP treatment, supporting the authors' hypothesis. Based on those results, the authors concluded that LF2 directly (or indirectly) phosphorylates the activation loop of CDKL5. I agree that the authors' hypothesis is one possibility, but there may be other explanations for the results. LF2 may phosphorylate CDKL5 at other positions than S162T164Y166, and the phosphorylation of the residue may inhibit the trypsin digest through the ionic bond with lysine/arginine. LF2 depletion or phosphatase treatment may block the phosphorylation and increase the efficiency of trypsin cleavage, which would result in increase in the intensity of the precursors. Besides, each of S162T164Y166 is highly phosphorylated in LF2 mutants, so it would not be easy to think that LF2 directly (or indirectly) phosphorylates the activation loop. I understand that figuring out what exactly is happening in those experiments is not trivial (the authors may possibly inject synthetic peptides that correspond to the trypsinized peptides that include activation loop with or without phosphorylation to prove the authors' hypothesis), and I do not think doing it is absolutely critical. Instead, please consider discussing alternative possibilities that could explain the results.

We thank Dr. Kanie for suggesting a very reasonable alternative hypothesis to explain our results. We actually had considered this possibility when trying to understand our data, but decided it was unlikely because peptides adjacent to the activation-loop peptide did not provide evidence for a missed cleavage. In our original paper we alluded to this when we stated that “Peptides immediately before and after the activation-loop peptide did not show this pattern; they were detected in similar amounts in all three strains (S2 Table).” However, we agree with Dr. Kanie that the alternative hypothesis should be clearly presented and discussed, and we have now done this:

“The above findings presented a conundrum – why were the activation-loop peptides not being detected in samples from control cells expressing CDKL5-GFP or CDKL5^K33R^-GFP even though they were readily detected when the protein was obtained from *lf2* cells? One possibility is that the LF2 kinase phosphorylates CDKL5 at a position that blocks trypsin cleavage so that the activation-loop peptide is not released from adjacent peptides. If this were the case, we would expect a longer peptide to be generated. R170 next to S168 and T169 would be the most likely blocked cleavage site. Failure to cleave at R170 would produce a peptide from aa155-173 but this peptide was not detected (Table S2). Similarly, there was no evidence for a missed cleavage involving R154 at the N-terminus of the activation-loop peptide. Therefore, failure to be cleaved is unlikely to account for the reduced amount of activation-loop peptide detected in CDKL5 or CDKL5K33R expressed in the presence of LF2.”

The data shown in Figure 6 is also complicated. The fact that CDKL5 3A (S162A, T164A, Y166A) mutant was not auto phosphorylated in vitro may suggest that the in vitro phosphorylation may occur at the S162/T164/Y166 residues. The failure of in vitro phosphorylation with the kinase-dead mutant (K33R) of CDKL5 confirmed that the in vitro phosphorylation in the CDKL5 was indeed mediated by CDKL5 itself (auto phosphorylation). These results may imply that the S162/T164/Y166 residues are the main sites of CDKL5 auto phosphorylation. However, this possibility conflicts with the data shown in figure 5, where the authors showed that the activation loop phosphorylation is likely NOT mediated by CDKL5 itself as there was no difference in the S162/T164/Y166 phosphorylation between wild-type and the kinase-dead mutant of CDKL5. Another possibility to explain the results is that the CDKL5 3A (S162A, T164A, Y166A) mutant lost its kinase activity. Perhaps, this is what the authors are thinking about. Figuring out what is exactly happening may require substantial amount of work, and that itself can be sufficient for a paper. Again, I do not think that is absolutely critical for this paper, but would like to ask the authors to explain their interpretations and discuss alternative possibilities in the main text. This would help readers to interpret the presented data correctly.

Dr. Kanie is correct that the result with CDKL5^S162A,T164A,Y166A^-GFP is not informative regarding specific sites that might be either autophosphorylated or phosphorylated by LF2, because the triple mutation would be expected to block either phosphorylation pathway. We have now changed the last sentences of the paragraph to:

“Thiophosphorylation was readily detected on CDKL5 but not on CDKL5^K33R^ (Fig 6) indicating that CDKL5 can autophosphorylate. No thiophosphorylation was detected on CDKL5^S162A,T164A,Y166A^ likely indicating that the kinase is unable to be activated, but this experiment cannot rule out that one or more of these residues is the target of thiophosphorylation.”

Other minor comments

(1)	Page 5 "CDKL5 is a serine/threonine protein kinase": I was initially confused with the fact that CDKL5 may autophosphorylate at tyrosine residue within the activation loop, as the authors initially described CDKL5 as a serine/threonine protein kinase. Based on the fact, this kinase should be considered as a dual-specificity kinase. I think it would reduce confusion if the authors clearly state the word "dual specificity kinase' early on in the paper.

Although Munoz et al. (2018) report that CDKL5 autophosphorylates Tyr171, UniProt (<https://www.uniprot.org/uniprotkb?query=cdkl5>) and many authors (Castano et al 2023 PMID 37162893; Katayama et al. 2020 PMID 32587608; Silvestre et al. 2024 PMID 38326557 ) still describe CDKL5 as a “serine/threonine” kinase. Rather than possibly confuse readers by specifying either “serine/threonine kinase” or “dual-specificity kinase,” we have simply removed “serine/threonine” from the description of CDKL5.

(2)	Figure 3A: Figure legends indicated that the authors stain the cells with CEP164, but I did not clearly see CEP164 in the Figure. Also, asterisk and arrowhead were not explained in the figure legend.

Thank you for catching this error. DAPI was in blue and has been adjusted to make it more visible in the composite image. Cells stained for Cep64 are shown in Fig 8.

(3)	Figure 3B and C: I initially got confused with the label "MS234, MS238, GP1192", as those labels were not explained in the figure legend. Referring to Table S8 in the Figure legend would be helpful. Similarly, throughout the paper, the authors use unique labels for Chlamydomonas lines (e.g., g1, 21gr), MEF lines (e.g., 26548), and plasmids (e.g., MS234). I appreciate that the authors listed what exactly they used, and think that it would help readers to easily figure out what exactly those labels are if the authors refer them to the corresponding tables (e.g., Table1, table S8) in the corresponding figure legends.

Plasmid names have been removed, and we have added a call out to Table S8 in the figure legend. Readers have now been referred to Table 1 in the legends of several figures beginning with Fig 2.

(4)	Figure 3B and 3C: It would be easier to understand what the 2A mutant is if the authors write the actual mutations (in this case, T183A and Y185A?)

This has been done. Similarly, CDKL5^3A^ has been changed to CDKL5^S162A,T164A,Y166A^.

(5)	Figure 4A: I feel dots are too small, and "K" and "*" marks are difficult to find. Please use bigger fonts.

This panel has been replaced with a larger snake diagram.

(6)	Figure 4B: Maybe I am missing, but I feel I could not find the methods for the CIP treatment of the flagella samples (I was able to find the methods for the CIP treatment of the mass spec samples shown in Figure 5B).

Methods for the CIP treatment of the samples for the phos-tag gels have been added to the Materials and Methods in the Western Blot subsection

“For CIP treatment, purified flagella samples were resuspended in HMDEK buffer (30 mM HEPES, pH 7.4, 5 mM MgSO_4_, 1 mM DTT, 0.5 mM EGTA, 25 mM KCl). Equal amounts of the same sample were either treated with or without Quick CIP (M0525S, New England Biolabs, Ipswich MA USA) in 1X rCutSmart buffer for 30 min at 37°C. The samples were heated at 80 °C for 2 minutes to inactivate CIP.”

(7)	Figure 5Ba: I did not understand why S162T164, S162Y166, and Y166 are omitted in this table.

S162T164, S162Y166, and Y166 were omitted from Table 5Ba because they were not detected in the MS analysis of the wild-type CDKL5 samples. Original Table 5Ba has been replaced with new Fig 5C, which includes these peptides but visually indicates that their levels were below 1E+5 or undetectable.

(8)	Page 31 line 6-page 32: The discussion in these pages are highly overlapped with the discussion written in the prior pages. Please consolidate the discussion, so that readers can take the most important findings of the paper.

The Discussion has been rewritten to be more concise and to eliminate repetition.

**Other minor revisions**

A minor change has been made to Fig 10B to start the Y axis at a ratio of 1.0 rather than 0.0. The values remain unchanged.
