# Supplementary material for: Activation of the ciliary kinase CDKL5 is mediated by the cyclin-dependent kinase CDK20/LF2 to control flagellar length
Source: PLoS Biol. 2025 Dec 12;23(12):e3003560. doi: 10.1371/journal.pbio.3003560 (PMC12711092; doi:10.1371/journal.pbio.3003560)
Supplement: Attachment — Submitted filename: Rebuttal_10_29_2025.docx [file pbio.3003560.s032.docx]

Reviewers' comments

Rev. 1:

In my original review of the study on CDKL5 by Pazour and colleagues I criticized the lack of clarity of their manuscript (major point 1), which made it largely inaccessible to the reader. This was particularly unfortunate given the clear and pressing need to make the not insubstantial community of clinical researchers working on Cdkl5 Deficiency Disorder aware of the potential ciliary connection of their disorder. In preparing this revision, the authors have extensively reworked the text, which has improved considerably in my opinion. The one recommendation I would still have here given the aforementioned need to raise awareness is to incorporate cilia into the title and use vertebrate nomenclature, perhaps: "Phosphorylation by LF2/CDK20 controls activity of the ciliary kinase CDKL5".

Title has been changed to incorporate the reviewer’s suggestions of including the word “ciliary” and the name of the mammalian ortholog.

New title: LF2/CDK20 phosphorylation of the activation loop regulates the ciliary kinase CDKL5

The revised text now more clearly elaborates the manuscript's novel findings and also discusses CDKL5 in the context of the wider CDKL kinase family, which appears to share a related function. Nevertheless, given that this was supposed to have been a major revision, I would have expected at least some attempt to address my second major point concerning a lack of mechanistic insight with additional experiments. I understand the authors do not wish to expand the scope of their present study and investigate the relationship between CDKL5 and other CDKL kinases. However, especially considering the authors have actually generated CDK20/CDKL5 double mutants, an analysis of ciliary lengths in those mutants compared to the individual single mutants as strongly suggested in my original review was entirely within its present scope and would have considerably strengthened the manuscript without being particularly demanding. To me this is a striking omission that should still be corrected.

We have added the data on the lengths of the Cdkl5 and Cdk20 mutants to Figure 8. Corresponding revisions have been made to the figure legend and the associated text.

Concerning the other, minor, points raised in my original review, these have been adequately addressed.

Rev. 3: Tomoharu Kanie

The authors addressed all my comments. I appreciate it.
